# CoVac501, a self-adjuvanting peptide vaccine conjugated with TLR7 agonists, against SARS-CoV-2 induces protective immunity

**DOI:** 10.1038/s41421-021-00370-2

**Published:** 2022-02-01

**Authors:** Yiru Long, Jianhua Sun, Tian-Zhang Song, Tingting Liu, Feng Tang, Xinxin Zhang, Longfei Ding, Yunqiu Miao, Weiliang Zhu, Xiaoyan Pan, Qi An, Mian Qin, Xiankun Tong, Xionghua Peng, Pan Yu, Peng Zhu, Jianqing Xu, Xiaoyan Zhang, Yachun Zhang, Datao Liu, Ben Chen, Huilin Chen, Leike Zhang, Gengfu Xiao, Jianping Zuo, Wei Tang, Ji Zhou, Heng Li, Zhijian Xu, Hong-Yi Zheng, Xin-Yan Long, Qiuping Qin, Yong Gan, Jin Ren, Wei Huang, Yong-Tang Zheng, Guangyi Jin, Likun Gong

**Affiliations:** 1 State Key Laboratory of Drug Research, Shanghai Institute of Materia Medica, Chinese Academy of Sciences, Shanghai, China; 2 University of Chinese Academy of Sciences, Beijing, China; 3 Key Laboratory of Animal Models and Human Disease Mechanisms of the Chinese Academy of Sciences, Kunming Institute of Zoology, Chinese Academy of Sciences, Kunming, Yunnan China; 4 Shanghai Public Health Clinical Center & Institutes of Biomedical Sciences, Fudan University Shanghai, China; 5 State Key Laboratory of Virology, Wuhan Institute of Virology, Chinese Academy of Sciences, Wuhan, Hubei China; 6 Shanghai King-Cell Biotechnology Co., Ltd, Shanghai, China; 7 Zhongshan Institute for Drug Discovery, Institutes of Drug Discovery and Development, Chinese Academy of Sciences, Zhongshan, Guangdong China; 8 Mabwell (Shanghai) Bioscience Co., Ltd, Shanghai, China; 9 School of Pharmaceutical Sciences, Shenzhen University Health Science Center, Shenzhen University, Shenzhen, Guangdong China; 10 International Cancer Center, Nation-Regional Engineering Lab for Synthetic Biology of Medicine, Shenzhen University, Shenzhen, Guangdong China; 11 School of Pharmaceutical Science and Technology, Hangzhou Institute for Advanced Study, University of Chinese Academy of Sciences, Hangzhou, Zhejiang China

**Keywords:** Immunology, Biological techniques

## Abstract

Safe, effective, and economical vaccines against severe acute respiratory syndrome coronavirus 2 (SARS-CoV-2) are needed to achieve adequate herd immunity and end the pandemic. We constructed a novel SARS-CoV-2 vaccine, CoVac501, which is a self-adjuvanting peptide vaccine conjugated with Toll-like receptor 7 (TLR7) agonists. The vaccine contains immunodominant peptides screened from the receptor-binding domain (RBD) and is fully chemically synthesized. It has been formulated in an optimized nanoemulsion formulation and is stable at 40 °C for 1 month. In non-human primates (NHPs), CoVac501 elicited high and persistent titers of protective neutralizing antibodies against multiple RBD mutations, SARS-CoV-2 original strain, and variants (B.1.1.7 and B.1.617.2). Specific peptides booster immunization against the B.1.351 variant has also been shown to be effective in improving protection against B.1.351. Meanwhile, CoVac501 elicited the increase of memory T cells, antigen-specific CD8^+^ T-cell responses, and Th1-biased CD4^+^ T-cell immune responses in NHPs. Notably, at an extremely high SARS-CoV-2 challenge dose of 1 × 10^7^ TCID_50_, CoVac501 provided near-complete protection for the upper and lower respiratory tracts of cynomolgus macaques.

## Introduction

The uncontrolled transmission and ongoing evolution of severe acute respiratory syndrome coronavirus 2 (SARS-CoV-2) lead to a requirement for safe, effective, quickly designable, and economical vaccines to rapidly respond to viral mutations and halt the epidemic of coronavirus disease 2019 (COVID-19)^1–5^. Multiple vaccines based on different technology platforms are now available and being used to combat the worldwide pandemic, including mRNA vaccines by BioNTech/Pfizer and Moderna, protein subunit vaccines by Novavax and Zhifei Longcom, inactivated vaccines by Sinopharm and SINOVAC, viral vector vaccines by AstraZeneca/University of Oxford and CanSino, etc.^6^.

Distinguished from these platforms, the peptide vaccine platform has unique advantages as an alternative approach to meet the challenges. The computer-based rational design allows peptide vaccines to be rapidly designed and respond to mutations based on critical epitopes of pathogens^7,8^. Moreover, peptide vaccines can be completely chemically synthesized on a large scale with low production costs, which can reduce the economic burden on public health.

However, the immunogenicity of peptides is relatively low^7,9^. Conjugating with innate immune agonists to form self-adjuvanting vaccine is a promising way to elicit specific antigen presentation and improve vaccination effects of peptide vaccines^10,11^. Toll-like receptor 7 (TLR7) is a natural immune pattern-recognition receptor localized on the endo/lysosome membrane^12^. TLR7 agonists are being developed in combination with various vaccines in our and others’ studies to facilitate the antigen presentation, activate innate immunity, promote antibody affinity maturation, and maintain long-lasting immune memory^13–16^.

We have developed a novel SARS-CoV-2 vaccine named CoVac501, which is a fully chemically synthesized and self-adjuvanting peptide vaccine conjugated with TLR7 agonists. In this study, we have optimized the vaccine formulation and evaluated the stability, immunogenicity, and protective efficacy of CoVac501 against SARS-CoV-2 original strain and variants.

## Results

### Prediction and screening of peptide candidates

The interaction between the receptor-binding domain (RBD) of spike (S) protein and angiotensin-converting enzyme 2 (ACE2) underlies SARS-CoV-2 entry into host cells^17,18^. Several studies have shown that the RBD is an immunodominant region in the S protein, and neutralizing antibodies (NAbs) in convalescent patients’ sera are mostly against the RBD^19–23^. Screening of NAbs epitopes from the RBD for immunization holds promise for inducing specific and protective humoral immune responses.

**Fig. 1:**
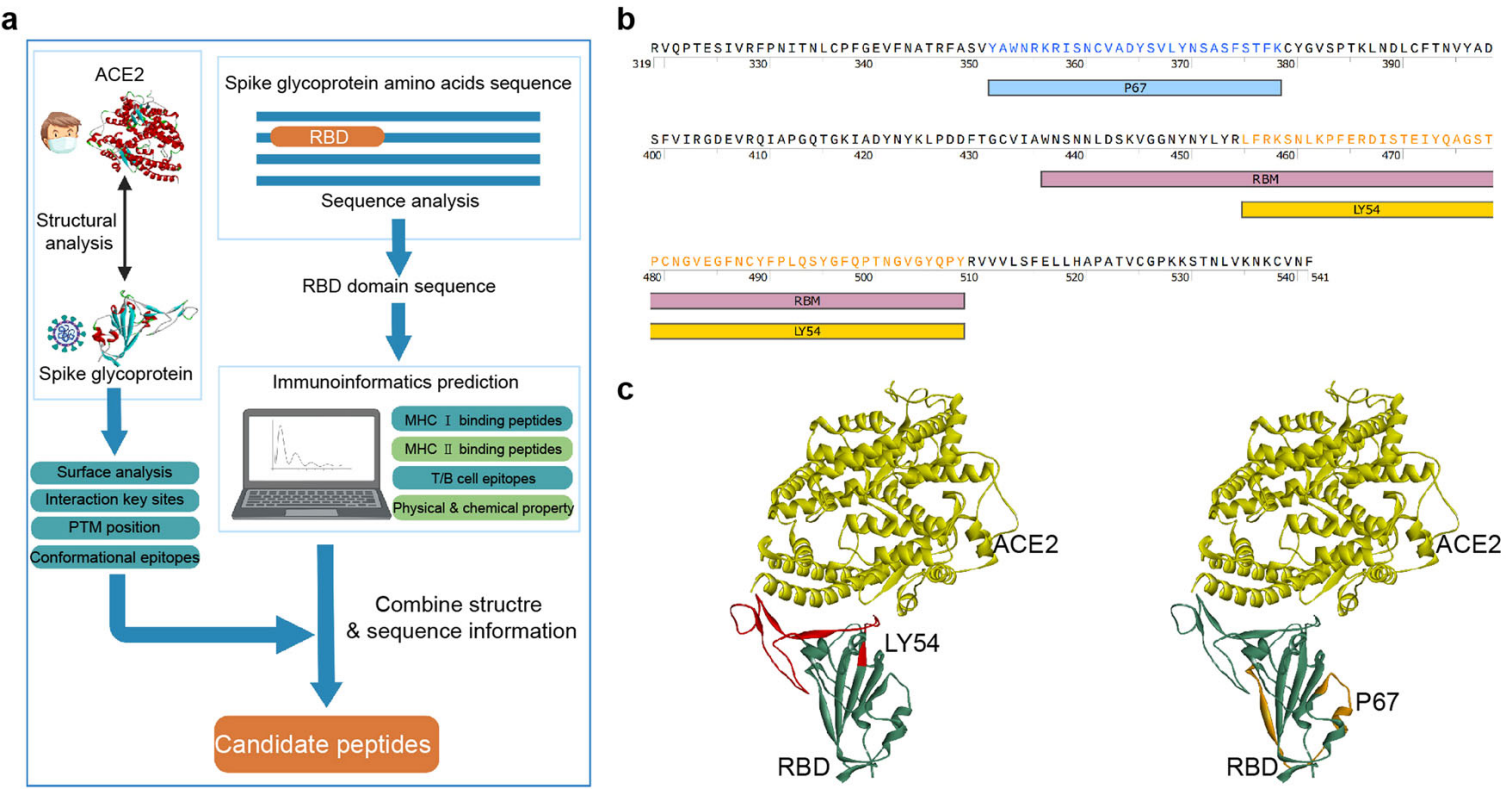
Identification of immunodominant candidate peptides. **a** The flowchart illustrates the prediction and screening process of the RBD immunodominant peptides. **b** The amino acid sequences of candidate peptides LY54 and P67. RBM receptor-binding motif. **c** The spatial localization of candidate peptides LY54 and P67 in the RBD. The crystal structure of the SARS-CoV-2 RBD bound to ACE2 was obtained from the Protein Data Bank (PDB; code 6M0J). The red color shows the location of LY54 and the orange color shows the location of P67.

Based on computer-aided vaccine design technology, we integrated the sequence and structural features of RBD to predict and screen several immunodominant peptide candidates (Fig. 1a). We predicted possible binding peptides for major human leukocyte antigen (HLA) class II molecules (Supplementary Fig. S1a, b) through NetMHCIIpan-4.0^24^. The RBD contains three immunodominant regions: residues around 345–380, 450–470, and 485–515. Consistently, these regions also contain potential HLA class I epitopes (Supplementary Fig. S1c) and B-cell linear epitopes (Supplementary Fig. S1d) predicted by NetMHCpan-4.1^24^ and BepiPred-2.0^25^. Considering the physicochemical properties, we selected LY54 (residues 455–508) and P67 (residues 351–378) as candidate peptides (Fig. 1b and Supplementary Fig. S1e). Notably, LY54 is positioned at the interaction interface between RBD and ACE2 (Fig. 1c). However, by predicting the possible binding peptides throughout the RBD region for mouse major histocompatibility complex (MHC) II molecules (Supplementary Fig. S2), it was observed that mouse MHC II molecules recognized LY54 and P67 much weaker than human MHC II molecules, suggesting potential species differences in the antigen epitopes of the RBD and the immunogenicity of LY54 and P67. Using a peptide solid-phase synthesis technique, we prepared LY54 and P67 with high purity.

### Preparation and identification of peptides conjugated with TLR7 agonists

**Fig. 2:**
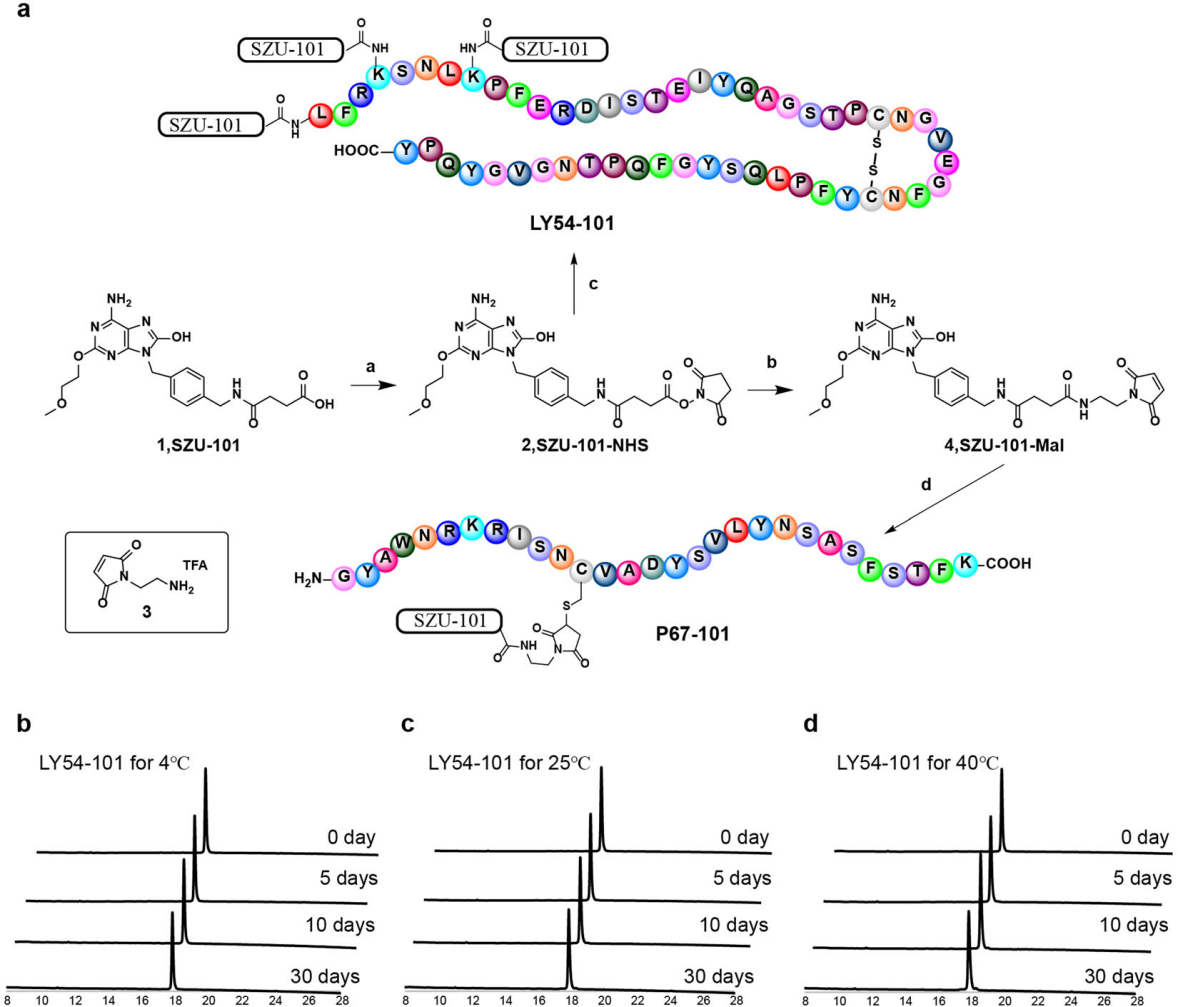
Full chemical synthesis and thermal stability analysis of self-adjuvanting peptides. **a** Synthesis of SZU-101 derivatives and SZU-101-peptide conjugations. Reaction conditions: a, EDCI/NHS, DMSO, 15 °C, overnight; b, 3, EDCI/NHS, DMSO, 15 °C, overnight; c, LY54, NaHCO_3_, DMF/H_2_O, room temperature (RT), 2 h; d, P67, NaHCO_3_, DMF/H_2_O, RT, 1 h. **b**–**d** HPLC profile of stability analysis of LY54–101 for 5 days, 10 days and, 30 days at 4 °C (**b**), 25 °C (**c**), and 40 °C (**d**). The horizontal axis represents the retention time (min), and the vertical axis represents the absorbance (mAU).

Through conjugation with TLR7 agonists, peptides can form self-adjuvanting vaccines, which will allow antigen-presenting cells (APCs) to simultaneously uptake peptide antigens and TLR7 agonists adjuvants to induce potent and specific antigen-specific immune responses^26^. We have developed a TLR7 agonist, SZU-101^27^, which has been applied in our previous studies to construct self-adjuvanting vaccines^13,14^. To conjugate LY54 and P67 with SZU-101, the N-Hydroxysuccinimide (NHS) ester group and the maleimide (Mal) group were covalently conjugated to the carboxyl group of SZU-101, respectively (Fig. 2a). LY54–101 was prepared by conjugating SZU-101-NHS with the leucine (residue 1) and lysine (residues 4 and 8) of LY54 (Fig. 2a). SZU-101-Mal was attached to the cysteine (residue 12) of P67 via a sulfhydryl group to form P67–101 (Fig. 2a). LY54–101 and P67–101 were purified and characterized by high-performance liquid chromatography (HPLC) and high-resolution mass spectrometry (HRMS) to achieve more than 95% purity (Supplementary Figs. S3–S8).

Considering that the thermal stability of vaccines can greatly affect the shipping and storage conditions, we tested the thermal stability of LY54–101 and P67–101. LY54–101 was placed at 4, 25, and 40 °C for 5, 10, and 30 days, respectively. HPLC identification results showed that no significant changes were found in the purity of LY54–101 at 4, 25, and even 40 °C for 30 days (Fig. 2b–d), suggesting that LY54–101 has outstanding thermal stability. Similar results were found for P67–101 at 40 °C for 41 days (Supplementary Fig. S9).

In summary, through a full chemical synthesis approach, we prepared thermostable peptides conjugated with TLR7 agonists as novel self-adjuvanting peptide vaccine candidates against SARS-CoV-2.

### Immunogenicity of LY54–101 and P67–101

After conjugating with TLR7 agonists, the synthetic peptides, LY54–101 and P67–101, possessed a potent immune-stimulating activity (Supplementary Fig. S10). To preliminarily evaluate the immunogenicity of LY54–101 and P67–101, C57BL/6 mice were injected with LY54–101 (50 μg) and P67–101 (50 μg) mixed with Titermax adjuvant on days 0, 7, and 14 for a total of three doses. The serum samples obtained 20 days after the third vaccination showed a strong and specific antibody response against RBD, with IgG-type antibody titers of up to 1:1,000,000 (Supplementary Fig. S11a). Notably, the serum samples obtained 56 days after the third vaccination still showed RBD-specific IgG-type antibody titers of up to 1:400,000 (Supplementary Fig. S11b). However, the antisera obtained from the mice exhibited ineffective ACE2-RBD blocking activity (Supplementary Fig. S11c), which was likely due to antibodies either generated from the non-neutralizing epitopes in LY54 and P67 or lacking adequate affinities for neutralizing activities. Consistent with our results, there are reports showing that linear peptides have difficulty in inducing neutralizing antibodies in mice^22,23^, which seemed relevant to the species differences that we found hereinbefore in the recognition of LY54 and P67 between human and murine MHC class II molecules. Thus, the immunogenicity of LY54–101 and P67–101 was further assessed in an NHP model. Cynomolgus monkeys were injected with antigens mixed with Titermax adjuvant for two doses on days 0 and 14 (Supplementary Fig. S11d). The sera of the LY54–101 + P67–101 group obtained 20 days post the second vaccination showed a strong and specific antibody response against RBD, with IgG-type binding antibody titers of up to 1:24,300 (Supplementary Fig. S11e) and ACE2-RBD blocking antibody titers of greater than 1:160 (Supplementary Fig. S11f). Besides, the LY54–101 + P67–101 group showed the best immunogenicity as compared to the LY54 + P67 and LY54 + P67 + SZU-101 groups. Notably, the sera from the LY54–101 + P67–101 group obtained 91 days after the second vaccination still showed RBD-specific IgG-type binding antibody titers of up to 1:24,300 (Supplementary Fig. S11g) and ACE2-RBD blocking antibody titers of greater than 1:320. (Supplementary Fig. S11h), suggesting that the self-adjuvanting peptide vaccine induced a potent and long-lasting humoral immune response. Furthermore, to compare the immunogenicity of our self-adjuvanting peptide and RBD protein, cynomolgus monkeys were injected with LY54–101 or RBD mixed with Titermax adjuvant for three doses on days 0, 14, and 28 (Supplementary Fig. S12a). Although RBD induced ninefold more RBD-specific IgG-type binding antibodies than LY54–101 (Supplementary Fig. S12b), the levels of ACE2-RBD blocking antibodies induced by the two immunogens were essentially comparable (Supplementary Fig. S12c), revealing the potential advantage of peptide vaccines for the enrichment of neutralizing antibody epitopes.

### Vaccine formulation optimization and in vivo delivery

Adjuvant formulations suitable for our self-adjuvanting peptide vaccine and available for clinical application are needed to replace Titermax. Nanoemulsion formulations can protect vaccine antigens, achieve slow release of the antigen and promote antigenic load of lymph nodes, and have been widely used in vaccine development, such as AS03^28,29^. We developed two oil-in-water nanoemulsion formulations (F1 and F2) and compared their effects with AS03 and Titermax for the in vivo delivery of self-adjuvanting peptides. F1 nanoemulsions are used to enhance the solubilization of self-adjuvanting peptides, while F2 nanoemulsions are used to improve the local retention of self-adjuvanting peptides.

**Fig. 3:**
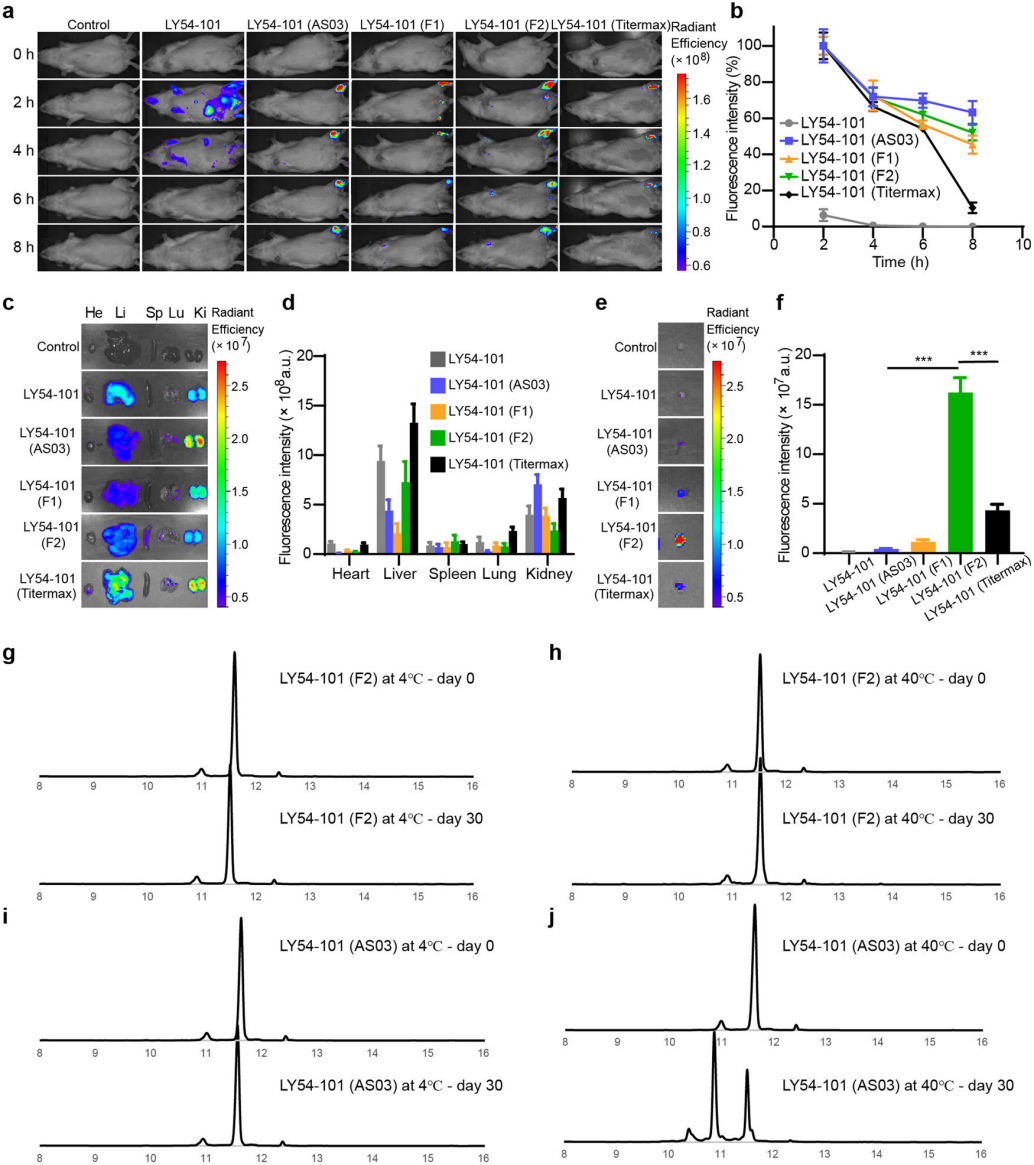
Biodistribution and retention of peptide vaccine nanoemulsions in vivo. **a** Real-time in vivo fluorescence images of rats after intramuscular injection of Cy5-labeled LY54–101 nanoemulsions. **b** Monitoring of the kinetics of LY54–101 nanoemulsion retention at injection sites within 8 h. (*n* = 3; means ± SD). **c**, **d** Biodistribution of Cy5-labeled LY54–101 nanoemulsions in main organs excised from rats at 8 h post injection. (*n* = 3; means ± SD). **c** Fluorescence images of organs. **d** Fluorescence intensity analysis. He, hearts; Li, livers; Sp, spleens; Lu, lungs; Ki, kidneys. **e**, **f** Fluorescence images (**e**) of lymph nodes excised at 8 h post injection and fluorescence intensity analysis (**f**) of Cy5-labeled LY54–101 nanoemulsions. (*n* = 3; means ± SD), ***P < 0.001 as determined by one-way ANOVA with multiple comparison tests. **g**–**j** HPLC profile of stability analysis of F2 nanoemulsion formulation (**g**, **h**) and AS03 nanoemulsion formulation (**i**, **j**) for LY54–101 at 4 °C or 40 °C for 30 days. The horizontal axis represents the retention time (min), and the vertical axis represents the absorbance (mAU).

To examine the retention of vaccine formulation at injection sites, several Cy5-labeled LY54–101 formulations were intramuscularly injected into the upper inner thigh of rats, and the fluorescence signal in inner thighs over 8 h was monitored using a live animal fluorescence imaging system (Fig. 3a). Free LY54–101 showed little fluorescence signal 4 h post injection, while LY54–101 nanoemulsions showed enhanced retention at injection sites (Fig. 3b). The F2 nanoemulsion displayed a slightly decreased fluorescence signal as compared to the AS03 nanoemulsion, with more than 50% of fluorescence intensity remaining at the injection sites (Fig. 3b).

In vivo biodistribution study results of different LY54–101 formulations indicated that LY54–101 was mainly metabolized through the liver and kidney (Fig. 3c, d). We then focused on the recruitment of different LY54–101 formulations in the lymph nodes. Little free LY54–101 was recruited in the lymph node, while nanoemulsions enhanced the recruitment of LY54–101 in the lymph node (Fig. 3e). Notably, the F2 nanoemulsion displayed the highest fluorescence intensity in lymph nodes among all vaccine formulations, including the Titermax (Fig. 3f). Similar results were found for the in vivo delivery of the F2 nanoemulsion formulation of P67–101 (Supplementary Fig. S13). Thus, we speculated that F2 nanoemulsions for self-adjuvanting peptides would obtain better immune efficacy than AS03 nanoemulsions.

We also tested the thermal stability of the vaccine formulations of LY54–101. The F2 nanoemulsion formulation and AS03 nanoemulsion formulation for LY54–101 were placed at 4 or 40 °C for 30 days, respectively. HPLC identification results showed no significant changes in the purity of LY54–101 in either the F2 nanoemulsion formulation or AS03 nanoemulsion formulation at 4 °C for 30 days (Fig. 3g, i). Notably, LY54–101 in the AS03 nanoemulsion formulation was degraded after 1 month at 40 °C, while LY54–101 in the F2 nanoemulsion formulation remained stable (Fig. 3h, j). In addition, the particle size of F2 nanoemulsion can be kept constant at 40 °C for 2 months (Supplementary Table S1). These results revealed that our nanoemulsion formulation F2 is of outstanding stability.

### Humoral immune responses in CoVac501-vaccinated cynomolgus macaques

**Fig. 4:**
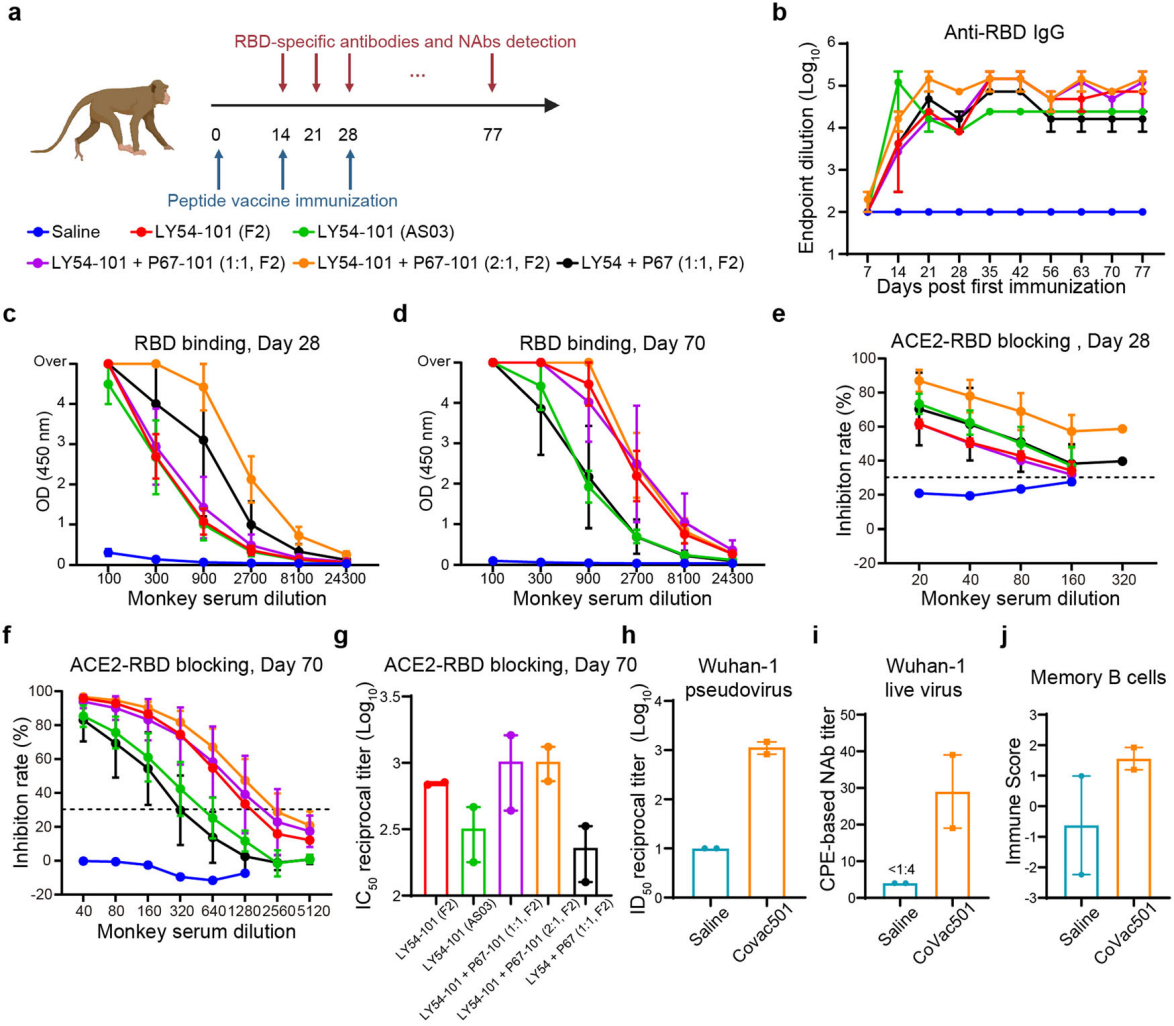
CoVac501 induced protective humoral immune responses in cynomolgus macaques. **a** Schematic diagram of immunization, sample collection, and detection in cynomolgus monkeys. Cynomolgus monkeys (*n* = 2) were immunized with peptide vaccines in the following groups: saline, LY54–101 (F2), LY54–101 (AS03), LY54–101 + P67–101 (1:1, F2), LY54–101 + P67–101 (2:1, F2), and LY54 + P67 (1:1, F2) for three doses at days 0, 14, and 28. **b**–**d** Sera were collected from the monkeys and the levels of RBD-specific antibody were tested for different serum dilutions using ELISA. **e**–**g** Sera were collected from monkeys 28 (**e**) and 70 (**f**, **g**) days after the first dose of vaccine and ACE2-RBD blocking antibodies were tested for different serum dilutions using ELISA. **h** The neutralization of sera from cynomolgus monkeys 63 days after the first dose of vaccine for SARS-CoV-2 pseudovirus (Wuhan-1). **i** The neutralization of sera from cynomolgus monkeys 56 days after the first dose of vaccine for live SARS-CoV-2 in vitro was tested by microdose CPE assay. **j** RNA sequencing results were analyzed for memory B cells in CoVac501-vaccinated monkeys. All data are presented as the means ± SEM.

To optimize the formulation composition (especially the ratio of LY54–101 and P67–101) and study the role of the TLR7 agonist in self-adjuvanting peptide vaccines, we vaccinated cynomolgus monkeys with the peptide vaccines in the following groups: saline, LY54–101 (F2), LY54–101 (AS03), LY54–101 + P67–101 (1:1, F2), LY54–101 + P67–101 (2:1, F2) and LY54 + P67 (1:1, F2) (Fig. 4a). Animals received peptide vaccines via the intramuscular route for three doses on days 0, 14, and 28, respectively. No clinical symptoms of the monkeys were observed after vaccination, and no significant changes in hematology parameters or body temperature were detected, demonstrating good safety for these vaccine formulations (Supplementary Tables S2 and S3).

Sera obtained on day 14 after the first dose already showed specific IgG-type binding antibody responses to the RBD, especially for the LY54–101 (AS03) group (Fig. 4b and Supplementary Fig. S14a). The titers of RBD-specific binding antibodies gradually increased with two booster immunizations from day 14 to day 35 and peaked between day 35 and day 42 (Fig. 4b–d and Supplementary Fig. S14a–f). By comparing the changes in IgG-type binding antibody levels between the groups, we were able to determine the optimal composition of the vaccine formulation. First, by comparing the LY54–101 (F2) and LY54–101 (AS03) groups, we found that the AS03 nanoemulsion could induce initial humoral immune responses more rapidly, but the antibody levels were also decreased fast, resulting in a much lower overall immune response than those induced by the F2 nanoemulsion, suggesting that F2 nanoemulsion is more suitable for adjuvanting peptide vaccines. Second, P67–101 further increased the production of specific antibodies to the RBD, and the optimal ratio of LY54–101 and P67–101 was 2:1. Third, by conjugating with the TLR7 agonist SZU-101, the peptide vaccines induced a stronger and longer-lasting humoral immune response. The titer of RBD-specific binding antibodies was maintained without detectable reduction to at least day 95 in the LY54–101 + P67–101 (2:1, F2) group (Supplementary Fig. S14g). Therefore, we identified LY54–101 + P67–101 (2:1, F2) as the final self-adjuvanting peptide vaccine formulation and named the product CoVac501.

The levels of ACE2-RBD blocking antibodies were high in all groups. Notably, the blocking antibody titers of the CoVac501 group were increased in magnitude after boosting and maintained at a stable level from day 63 to day 70, which was eventually up to 1:2560 or a mean 50% inhibitory concentration (IC_50_) of 1:1024 (Fig. 4e–g and Supplementary Fig. S14h–j).

Further, serum neutralization titers for SARS-CoV-2 pseudovirus and live virus were determined. CoVac501 induced pseudovirus neutralization with a mean 50% infectious dose (ID_50_) of 1:1150 in cynomolgus monkeys on day 63 (Fig. 4h and Supplementary Fig. S16a). Sera from CoVac501-vaccinated monkeys had a neutralization titer for authentic virus up to 1:39 at 28 days after the third immunization (Fig. 4i) detected by microdose cytopathogenic efficiency (CPE) assays. In addition, to further characterize the humoral immunity alterations in monkeys vaccinated with CoVac501, peripheral blood mononuclear cells (PBMCs) were isolated from monkeys 24 h after the third immunization and processed for RNA sequencing. We analyzed the RNA sequencing results through xCell^30^ and found that CoVac501 induced a higher level of memory B cells in vaccinated monkeys (Fig. 4j).

Furthermore, we monitored the immunization effect of four different dose groups of vaccine formulations at optimized antigen ratios (Supplementary Fig. S15). All eight vaccinated monkeys produced high levels of RBD-specific and protective antibodies, even in the lowest dose group (0.08 mg LY54–101 + 0.04 mg P67–101). These results indicated that CoVac501 could induce a robust and durable protective humoral immune response.

### T-cell immune responses in CoVac501-vaccinated cynomolgus macaques

**Fig. 5:**
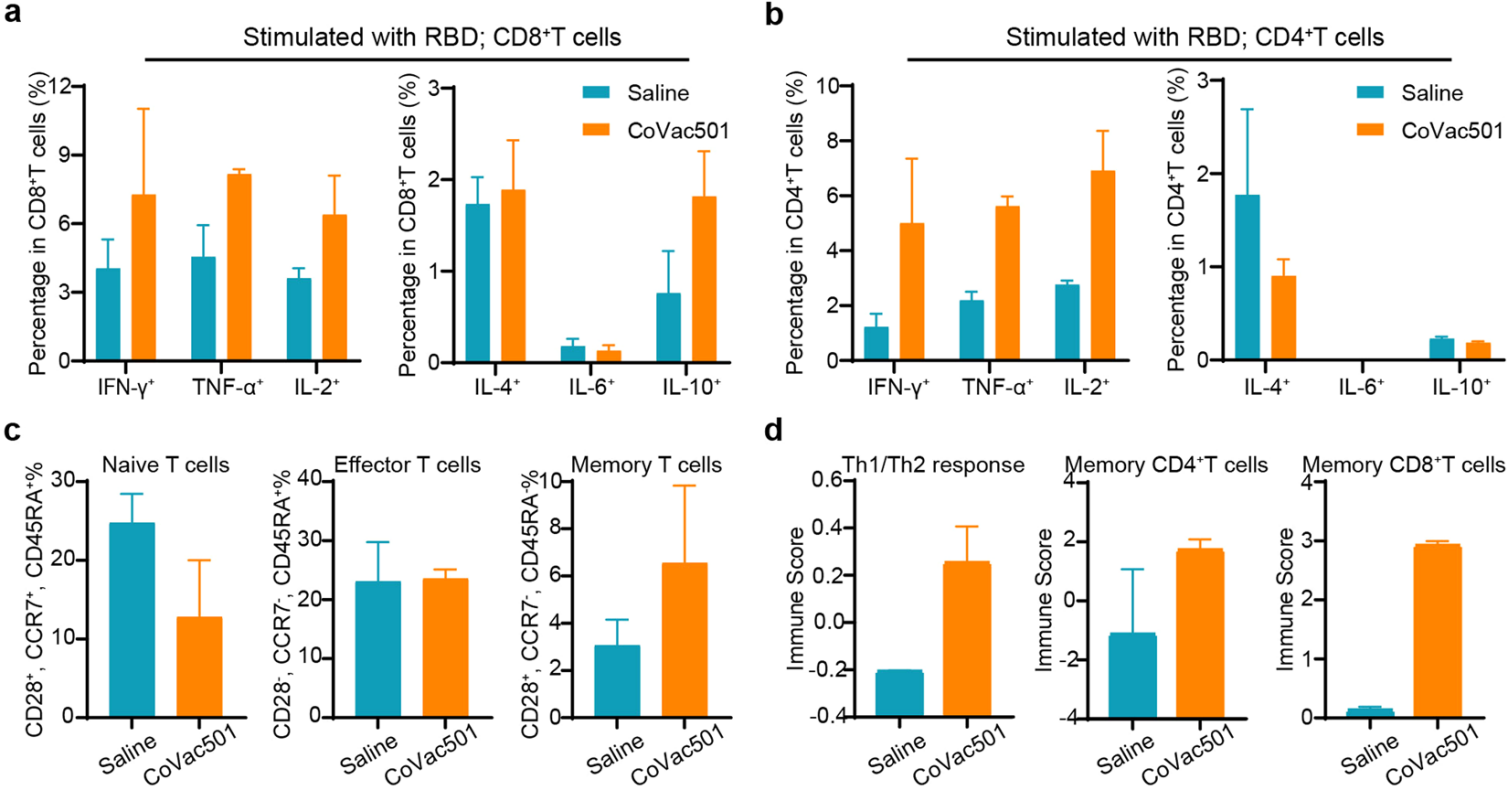
CoVac501 induced Th1-baised responses and T-cell immune memory in cynomolgus macaques. **a**, **b** PBMCs were collected from the monkeys 70 days after the first dose of vaccine and IFN-γ^+^, TNF-α^+^, IL-2^+^, IL-4^+^, IL-6^+^, and IL-10^+^ intracellular cytokine staining assays by flow cytometry for CD8^+^ T (**a**) and CD4^+^ T (**b**) cells in response to RBD after 8 h antigen stimulation. **c** PBMCs were collected from monkeys 42 days after the first dose of vaccine and naive T cells (CD28^+^, CCR7^+^ and CD45RA^+^), effector T cells (CD28 ^–^, CCR7^–^, and CD45RA^+^) and memory T cells (CD28^+^, CCR7^–^, and CD45RA^–^) were tested by flow cytometry. **d** RNA sequencing results were analyzed for immune cell levels and immune processes through the xCell or Molecular Signatures Database. All data are presented as the means ± SEM.

We also observed cellular immune responses in CoVac501**-**vaccinated monkeys. T cells of vaccinated monkeys (on day 28 and day 42) were quantified by flow cytometry. CoVac501**-**vaccinated monkeys had fewer naive T cells (CD28^+^, CCR7^+^, and CD45RA^+^) and more memory T cells (CD28^+^, CCR7^–^, and CD45RA^–^), suggesting that CoVac501 vaccination promoted T-cell activation and T-cell immune memory in cynomolgus monkeys (Fig. 5c and Supplementary Fig. S17a). In addition, PBMCs of vaccinated monkeys were stimulated in vitro with either LY54 peptides or RBD. Intracellular cytokine staining assays showed that CoVac501 induced an RBD or LY54-specific CD8^+^ T-cell responses (Fig. 5a and Supplementary Fig. S17b). Meanwhile, CoVac501 induced an increase in IFN-γ^+^, TNF-α^+^, and IL-2^+^CD4^+^ T cells without affecting the levels of IL-4^+^, IL-6^+^, and IL-10^+^ CD4^+^ T cells, indicating that CoVac501 induced Th1-biased immune responses in vaccinated monkeys (Fig. 5b and Supplementary Fig. S17c).

Based on analysis of RNA sequencing results, we also found Th1-biased immune responses and improved memory T-cell levels (Fig. 5d). In addition, CoVac501 promoted the activation of innate immunity, increased plasmacytoid DCs (pDCs), and decreased immature DCs (iDCs) in PBMCs (Supplementary Fig. S18), which may attribute to TLR7 agonists.

### Protective efficacy against SARS-CoV-2 challenge in cynomolgus macaques

**Fig. 6:**
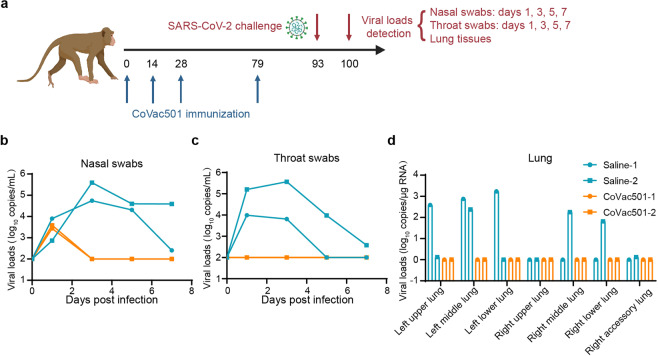
Protective efficacy against SARS-CoV-2 challenge in cynomolgus macaques. **a** Schematic diagram of immunization, SARS-CoV-2 challenge, sample collection, and detection in cynomolgus monkeys. Cynomolgus monkeys (*n* = 2) were challenged with a SARS-CoV-2 virus dose of 1 × 10^7^ TCID_50_ (20% nostril and 80% trachea) 14 days after a booster immunization on day 79. Nasal and throat swabs were collected 1, 3, 5, and 7 days after the challenge. The viral load in the lung tissues was determined seven days after the challenge. **b** Viral load detection for nasal swab samples during the challenge. **c** Viral load detection for throat swab samples during the challenge. **d** Viral load detection for left and right lung lubes after challenge.

Cynomolgus monkeys were challenged with an extremely high SARS-CoV-2 virus dose of 1 × 10^7^ TCID_50_ (50% tissue culture infective dose) 14 days after a booster immunization on day 79 to assess the protective effect of CoVac501 (Fig. 6a). We measured viral loads during the challenge process through nasal and throat swabs. Monkeys with saline administration had high viral load levels in nasal and throat swab samples as early as 1 day post exposure, with a mean peak of 5.17 (log_10_ RNA copies/mL) in nasal swab samples and a mean peak of 4.89 (log_10_ RNA copies/mL) in throat swab samples (Fig. 6b, c). Viral RNA was only observed at 1 day post exposure in the vaccine groups (Fig. 6b, c). Viral loads were undetectable in monkeys vaccinated with CoVac501 from day 3 to day 7 post exposure (Fig. 6b, c). After the challenge, viral loads in lung tissues were assessed. No monkeys in the CoVac501 group had a detectable viral load in any lung lobe (Fig. 6d), while a high viral load was detected in all left and right lung lobes in the saline group (Fig. 6d). These results demonstrated that CoVac501 could protect the upper and lower respiratory tracts of cynomolgus macaques from infection.

### Humoral immunity induced by CoVac501 against SARS-CoV-2 variants

**Fig. 7:**
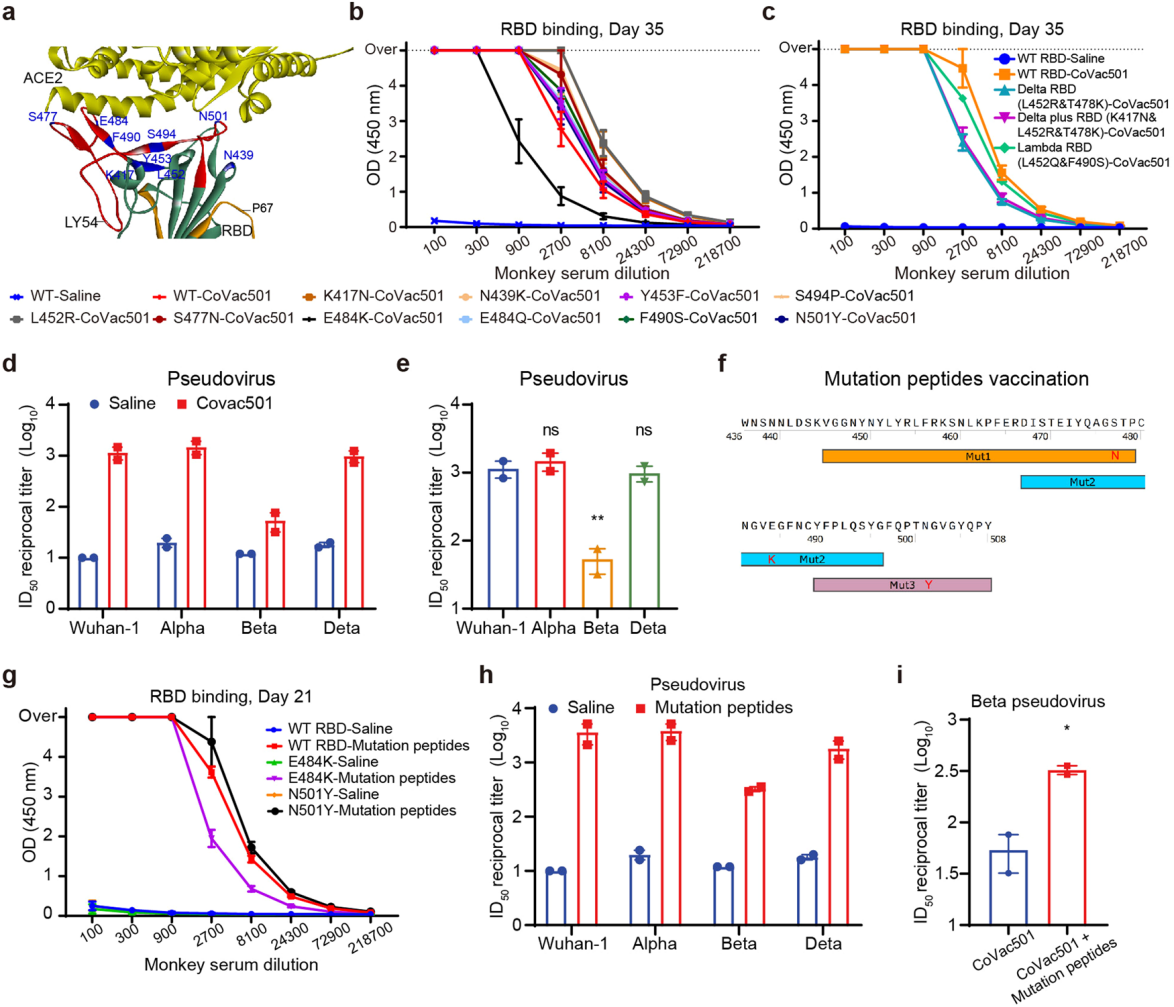
CoVac501 induced humoral immune responses against SARS-CoV-2 variants. **a** Positioning of K417N, N439K, L452R, Y453F, S477N, E484K, E484Q, F490S, S494P, and N501Y in RBD. **b**, **c** Cynomolgus monkeys (*n* = 2) were immunized with saline or CoVac501 for three doses at days 0, 14, and 28. Sera were collected from the monkeys 35 days after the first dose of vaccine and the levels of RBD single mutations (**b**) and combined mutations (**c**) binding antibodies were tested for different serum dilutions using ELISA. **d**, **e** The neutralization of sera from cynomolgus monkeys 63 days after the first dose of vaccine for SARS-CoV-2 original strain and variants (Alpha, Beta, and Delta) by the pseudovirus test. ID_50_ reciprocal titers were compared to the saline group (**d**) or between variants (**e**). **f** The graph shows the amino acid sequences of mutation peptides. **g** Cynomolgus monkeys (*n* = 2) were immunized with saline or mutation peptides for two doses at days 0 and 14. Sera were collected from the monkeys 21 days after the first dose of vaccine and the levels of RBD mutations (E484K and N501Y) binding antibodies were tested for different serum dilutions using ELISA. **h**, **i** The neutralization of sera from cynomolgus monkeys 21 days after the first dose of mutation peptides for SARS-CoV-2 original strain and variants (Alpha, Beta, and Delta) by the pseudovirus test. ID_50_ reciprocal titers were compared to the saline group (**h**) or to the CoVac501 group for Beta pseudovirus (**i**). **P < 0.01; *P < 0.05; ns, not significant as determined by unpaired *t-*test. All data are presented as the means ± SEM.

SARS-CoV-2 variants showed immune escape from existing vaccines^31–34^. Mutations in amino acid residues of the RBD may affect the effectiveness of existing vaccines and neutralizing antibodies^35^. We, therefore, tested changes in antibody titers of sera from CoVac501-vaccinated cynomolgus monkeys against ten mutant RBD proteins (K417N, N439K, L452R, Y453F, S477N, E484K, E484Q, F490S, S494P, and N501Y) compared to wild-type (WT) RBD (Fig. 7a). Monkey sera were obtained on days 35 and 70 after the first dose of CoVac501. The results showed that RBD-specific antibody levels were not affected by mutations of K417N, N439K, L452R, Y453F, S477N, E484Q, F490S, S494P, and N501Y of RBD (Fig. 7b and Supplementary Fig. S19a), suggesting that the effect of CoVac501 against the B.1.1.7 variants (Alpha) and B.1.617.2 variants (Delta) may not be affected by those mutations of the virus. Moreover, mutations as L452R&T478K (Delta), K417N&L452R&T478K (Delta plus), and L452Q&F490S (Lambda) did not affect the level of RBD-specific binding antibodies (Fig. 7c and Supplementary Fig. S19b). However, the binding antibody levels against RBD from the E484K mutation were threefold lower than those against RBD from the original virus (Wuhan-1; Fig. 7b and Supplementary Fig. S19a). We also tested the serum (day 63) neutralization titers for B.1.1.7, B.1.617.2, and B.1.351 pseudoviruses. Results showed that CoVac501 induced B.1.1.7 pseudovirus neutralization with a mean ID_50_ of 1:1490, indicating the efficacy of CoVac501 was not affected by B.1.1.7 variants (Fig. 7d, e and Supplementary Fig. S16b). For the most dominant variant so far, B.1.617.2 (Delta), CoVac501 induced B.1.617.2 pseudovirus neutralization with a mean ID_50_ of 1:986, indicating that the efficacy of CoVac501 was not affected by B.1.617.2 variants (Fig. 7d, e and Supplementary Fig. S16d). Compared to the mean ID_50_ titer of convalescent patient sera from our previous work^36^, the neutralizing activity of the antiserum of monkeys vaccinated with CoVac501 was comparable to that of convalescent patient sera. However, the protective effect of CoVac501 was notably decreased for neutralizing the B.1.351 (Beta) variant (Fig. 7d, e and Supplementary Fig. S16c).

Therefore, we designed and synthesized three mutant peptides with S477N, E484K, and N501Y, respectively (Fig. 7f). After a booster immunization of mutant peptides for CoVac501-vaccinated monkeys, sera were obtained to detect antibodies against RBD mutants. Results showed that the titers of binding antibodies against the RBD with E484K were equal to those against wild-type RBD (Fig. 7g), suggesting the CoVac501 supplemented with mutant peptides can provide protection against variants with the E484K mutation. Then we tested the serum (day 21) neutralization titers for B.1.351 pseudovirus. Mutant peptides induced B.1.351 pseudovirus neutralization with a mean ID_50_ of 1:324 (Fig. 7h, I and Supplementary Fig. S20c), indicating that CoVac501 supplemented with mutant peptides can provide potent protection against B.1.351. Meanwhile, mutant peptides induced enhanced neutralizing activity against Wuhan-1 pseudovirus (mean ID_50_ of 1:3614; Fig. 7h and Supplementary Fig. S20a), B.1.1.7 pseudovirus (mean ID_50_ of 1:3824; Fig. 7h and Supplementary Fig. S20b) and B.1.617.2 pseudovirus (mean ID_50_ of 1:1816; Fig. 7h and Supplementary Fig. S20d).

## Discussion

The self-adjuvanting peptide vaccine conjugated with TLR7 agonists is a novel form of vaccine that has the potential to be applied for COVID-19 or other infectious diseases and can serve as a rapid response strategic reserve for future outbreaks.

CoVac501 is an epitope-based peptide vaccine. Peptide vaccines can achieve rapid design for antigenic proteins through computer-aided vaccine design technology^8^. In addition, peptides can be efficiently and economically produced on a large scale by full chemical synthesis, allowing peptide vaccines to respond rapidly to pandemics. Notably, peptide vaccines can be modified to provide fast responses to pathogen mutations. Initially, CoVac501 induced limited antibody levels against the E484K RBD mutant and had limited protection against infection by the B.1.351 variant. This may be due to the E484K mutation leading to a local conformational destabilization and rearrangement of the loop region, which increases the binding affinity between RBD and ACE2 and decreases its affinity for vaccine-induced neutralizing antibodies^37,38^. After booster immunizations of the modified peptides, the monkey sera could provide stronger protection against the E484K RBD mutant and neutralize B.1.351. Moreover, considering the problem of immune imprinting, peptide vaccines for booster immunization can induce more mutant-specific antibodies against variants rather than against conserved epitopes^39^. The main components and vaccine formulation of CoVac501 all have outstanding thermal stability at 40 °C, which makes the storage and transportation of the vaccine very convenient. Therefore, our peptide vaccines are considered to be cost-effective and suitable for mass production.

CoVac501 contains two RBD-related peptides (LY54 and P67), which can elicit neutralizing humoral immune responses. Several studies on SARS-CoV-2 NAb development and analysis have shown that NAbs against the RBD can be divided into four classes that recognize the four corners of the RBD^19,40–45^. LY54 is in the region recognized by Class 1 and Class 2 antibodies that directly overlap with the ACE2-binding sites. P67 is in the region recognized by Class 3 antibodies, which is distal from the ACE2-binding site, and antibodies that recognize this region such as CR3022 can show neutralization through destroying the prefusion spike conformation^46^. In addition to these conformation or neutralizing antibody epitopes, previous studies have identified residues 370–394^22^, 450–469^22^ (or 455–469^23^) and 475–499^23^ (or 480–499^22^, 487–498^47^) as linear epitopes in the RBD region with a certain ability to elicit neutralizing antibodies. LY54 and P67 contain or have cross-coverage with these epitopes. All of these epitope studies further support the rationality of our vaccine design. On the other hand, as the study of NAb epitopes of SARS-CoV-2 progresses, introductions of new antigenic peptides into CoVac501 may further improve the immune effect of the vaccine, such as the N3 and N5 loops of the N-terminal domain (NTD) of the S protein^48,49^.

Although up to 1:640 (mean IC_50_ of 1:422) of NAb titers were induced by immunization with LY54 and P67, the immune effect lasted for a much shorter duration than LY54–101 and P67–101. CoVac501 induced a sustained increase in NAb titers and maintained a durable humoral immune response, which may be attributed to TLR7 agonists for promoting antibody affinity maturation and immune memory formation^16,50^. Interestingly, the genome of SARS-CoV-2 is linear single-stranded positive-sense RNA (ssRNA), which is recognized by TLR7 in innate immune cells^17,51,52^. Studies have shown that TLR7 expression levels and activity are lower in males, the elderly, or people with underlying diseases such as obesity, who are more susceptible populations to SARS-CoV-2^53,54^, suggesting a potential association between susceptibility to SARS-CoV-2 and TLR7 expression levels and activities. Adequate activation of TLR7 may also provide protection to susceptible individuals^55^.

Our present study still has some limitations that will be addressed in further researches. First, the specific role and mechanism of action of TLR7 agonist SZU-101 in the immune effect of CoVac501 remains to be investigated, especially how TLR7 agonists promote antibody affinity maturation and immune memory formation in humoral immunity in vivo. Second, the components and involved mechanism related to lymph node aggregation and better in vivo immune effect of the peptide vaccine caused by F2 nanoemulsion remains to be elucidated. Third, we need to further explore the advantage of CoVac501 as a thermostable vaccine candidate for intranasal immunization in the hope to block viral invasion at respiratory mucosa through IgA-mediated mucosal immunity^56^.

In conclusion, we have developed a novel SARS-CoV-2 vaccine, CoVac501, a fully chemically synthesized and self-adjuvanting peptide vaccine conjugated with TLR7 agonists. CoVac501 has an optimized nanoemulsion formulation and outstanding thermal stability suitable for both intranasal and intramuscular immunization. CoVac501 can induce high-level, protective, and long-lasting neutralizing humoral immune responses against SARS-CoV-2 original strain or variants and Th1-biased T-cell immune responses in NHPs. In addition, CoVac501 provided potent protection for cynomolgus macaques against challenges from very high doses of SARS-CoV-2. We believe that CoVac501 holds great potential for further clinical development and application.

## Materials and methods

### Animals and study design

Mouse care and experiments were performed at Shenzhen University, using protocols approved by the Institutional Laboratory Animal Care and Use Committee (IACUC). Female C57BL/6 mice (*n* = 5) were injected intramuscularly with LY54–101 (50 μg) and P67–101 (50 μg) mixed with Titermax adjuvant (Sigma) or saline for three doses at days 0, 7, and 14. Sera were collected at regular intervals for detection of the RBD-specific binding antibodies and NAbs.

Cynomolgus monkey husbandry and studies were conducted in an Association for Assessment and Accreditation of Laboratory Animal Care International (AAALAC) accredited Good Laboratory Practice (GLP) facility. In the first immunogenicity evaluation study of peptide vaccines in NHPs, cynomolgus monkeys (*n* = 2) were injected intramuscularly in the following groups: LY54–101 + P67–101 (1 mg + 1 mg), LY54 + P67 (1 mg + 1 mg) and LY54 + P67 + SZU-101 (1 mg + 1 mg + 500 μg) mixed with Titermax adjuvant or saline for two doses at days 0 and 14. Sera were collected at regular intervals. To compare the immunogenicity of LY54–101 and RBD, cynomolgus monkeys (*n* = 2) were injected intramuscularly in the following groups: LY54–101 (200 μg) and RBD (200 μg) mixed with Titermax adjuvant or saline for three doses at days 0, 14, and 28. In the immunogenicity evaluation study of vaccine formulations, we vaccinated cynomolgus monkeys with peptide vaccines in the following groups: saline, LY54–101 (2 mg, F2), LY54–101 (2 mg, AS03), LY54–101 + P67–101 (1:1, 2 mg + 2 mg, F2), LY54–101 + P67–101 (2:1, 2 mg + 1 mg, F2) and LY54 + P67 (1:1, 2 mg + 2 mg, F2) for three doses at days 0, 14, and 28. In the multi-dose evaluation study, we vaccinated cynomolgus monkeys with peptide vaccines in the following groups: saline, 0.08 mg LY54–101 + 0.04 mg P67–101, 0.4 mg LY54–101 + 0.2 mg P67–101, 0.8 mg LY54–101 + 0.4 mg P67–101, 1.6 mg LY54–101 + 0.8 mg P67–101, for three doses at days 0, 14, and 28. The preparation of vaccine nanoemulsions was performed as described below. Sera and PBMCs were collected at regular intervals for antibody detection and immune-response assays. The immunization procedure for mutant peptides (0.175 mg of Mut1, 0.15 mg of Mut2, and 0.1 mg of Mut3; F2 nanoemulsions) was consistent with that described above. In SARS-CoV-2 challenge assay, experiments were performed in the Kunming National High-level Biosafety Research Center for Non-human Primates, Center for Biosafety Mega-Science, Kunming Institute of Zoology, Chinese Academy of Sciences. The monkeys in the group above (saline and LY54–101 + P67–101 (2:1, 2 mg + 1 mg, F2)) were challenged with a SARS-CoV-2 virus (SARS-CoV-2 strain 107, NMDC000HUI) dose of 1 × 10^7^ TCID_50_ (20% nostril and 80% trachea) 14 days after a booster immunization on day 79. Nasal and throat swabs were collected 0, 1, 3, 5, and 7 days after the challenge and used to determine the viral load. Seven days after the challenge, all cynomolgus monkeys were euthanized. The viral load in the lung tissues was determined.

### Prediction of immunodominant peptide epitopes

The amino acid sequence of the SARS-CoV-2 spike protein was obtained from the UniProt database (P0DTC2). The sequence of the RBD was extracted from residues 319 to 541 of the spike protein. The human and mouse MHC class II-binding peptides of RBD for major human or mouse MHC class II types were predicted by NetMHCIIpan-4.0. The HLA class I-binding peptides of RBD for major HLA class I types were predicted by NetMHCpan-4.1. B-cell linear epitopes of RBD were predicted by BepiPred-2.0. Parameter settings are referred to the software recommendations. Crystal structure of SARS-CoV-2 RBD bound with ACE2 was obtained from the Protein Data Bank (PDB; code 6M0J)^18^. The structures are shown with the Discovery studio 2016 client.

### Synthesis of SZU-101 derivatives and SZU-101-peptide conjugates

SZU-101 was synthesized as previously reported^27^. EDCI, NHS and 1-(2-Aminoethyl)-1H-pyrrole-2,5-dione 2,2,2-trifluoroacetate were purchased from Bidepharm. DMF, DMSO and NaHCO_3_ were purchased from Sinopharm. Trifluoroacetic acid (TFA) and acetonitrile (ACN) were purchased from J&K. Peptides LY54 and P67 were synthesized by JYMedtech. All chemicals and solvents were used without further purification unless indicated. ^1^H and ^13^C NMR spectra were recorded on a 400, 500, or 600 MHz instrument. HPLC was performed on a Thermo U3000 instrument equipped with a C18 column (Thermo, Acclaim^TM^ 120, 5 μm, 4.6 × 250 mm). Compounds were purified by C18 preparative column (Waters, OBD, 5 μm, 10 × 250 mm) with proper eluent gradient. High-resolution mass spectrometry (HRMS) was performed on a Waters Xevo G2-XS QTof spectrometer. Synthesis of SZU-101-NHS was performed as below. SZU-101 (60 mg, 0.135 mmol, 1.0 eq), EDCI (64.76 mg, 0.34 mmol, 2.5 eq) and NHS (105.65 mg, 0.92 mmol, 6.75 eq) were dissolved in 1.5 mL DMSO and the solution was stirred at 15 °C. The reaction was monitored by analytical HPLC. After 14 h, the reaction mixture was poured into water and the product SZU-101-NHS was precipitated as a white solid from the solution. After three repeated washing with water, SZU-101-NHS was obtained as a white powder after lyophilization and was used without further purification. Synthesis of SZU-101-Mal was performed as below. SZU-101 (60 mg, 0.135 mmol, 1.0 eq), EDCI (64.76 mg, 0.34 mmol, 2.5 eq) and NHS (105.65 mg, 0.92 mmol, 6.75 eq) were dissolved in 1.5 mL DMSO. The solution was stirred at 15 °C and monitored by analytical HPLC. After 14 h, compound 3 (69 mg, 0.27 mmol, 2.0 eq) and 338 μL of 1 M NaHCO_3_ were added into the reaction mixture. The reaction was stirred at RT for another one hour before being purified by preparative C18 column and the product was obtained as a white powder after lyophilization. Synthesis of LY54–101 was performed as below. Peptide LY54 (816 mg, 0.133 mmol, 1.0 eq) was dissolved in a solution of DMF (20 mL) and water (5 mL). SZU-101-NHS (360.45 mg, 5.0 eq.) was added to the above solution, and a final concentration of 20–25 mM NaHCO_3_ was added. The reaction mixture was incubated at room temperature for 2 h and monitored by reverse HPLC. The product was purified by a preparative C18 column and obtained as a white powder after lyophilization. Yield, 820 mg, 83.33%. HRMS, calculated for C_339_H_468_N_88_O_98_S_2_, [M + 5H]^5+^ 1481.6835, found, 1481.6805; [M + 6H]^6+^ 1234.9042, found, 1234.8986; [M + 7H]^7+^ 1058.6333, found, 1058.6230. Synthesis of P67–101 was performed as below. Peptide P67 (160 mg, 1.0 eq) was dissolved in DMF (6.0 mL) and water (4.0 mL), and purified SZU-101-Mal (35.2 mg, 1.3 eq) was added to the above solution. A final concentration of 30–35 mM NaHCO_3_ was added to the reaction mixture. The reaction mixture was incubated at room temperature for 1 h before being purified by a preparative C18 column. The product was obtained as a white powder after lyophilization. Yield 69.52%. HRMS, calculated for C_177_H_253_N_49_O_51_S, [M + 4H]^4+^ 979.2186, found 979.2466; [M + 5H]^5+^ 783.5764, found 783.5913; [M + 6H]^6+^ 653.1483, found 653.1462.

### Thermal stability analysis

Taking the stability analysis of LY54–101 as an example, a certain amount (0.3–0.8 mg) of the conjugate was weighed into bottles. All bottles containing samples were separated into three groups and stored at 4, 25, or 40 °C, respectively. One bottle of each group was taken out at day 0, day 5, day 10, and day 30 for stability analysis. DMSO was added to the bottles to dissolve the samples, resulting in a concentration of 0.5 mg/mL. For HPLC analysis, 10 μL of each aliquot was injected and the column was eluted by a linear gradient of 2%–90% acetonitrile in 30 min at 1.0 mL/min, 40 °C.

### Splenocyte-stimulation assay

The spleen was taken out from the cynomolgus monkey under aseptic conditions. Single-cell suspensions of splenocytes were obtained by grinding spleen tissues in RPMI1640 medium with a syringe rubber pad, incubating with erythrocyte lysis buffer (Yeasen), and then filtering through a 70-μm nylon cell filter. Splenocytes (100 μL, 1 × 10^6^ cells/mL) were added to 96-well plates. Splenocytes were stimulated with SZU-101 (5 μM), LY54–101 (5 μM), LY54 (5 μM), P67 (5 μM), and P67–101 (5 μM) for 24 h. The detection of IFN-γ and TNF-α was performed using V-Plex Proinflammatory Panel 1 Human Kits (Meso Scale Diagnostics). Results were read and analyzed on a QuickPlex SQ120 (Meso Scale Diagnostics).

### Preparation and characterization of vaccine nanoemulsions

Peptide vaccines were dissolved in squalene (2.1%) and alpha-tocopherol (2.4%) to form a homogeneous oil phase, which accounted for 4.5% by volume in a nanoemulsion. Tween-80 as an emulsifier was dissolved in the oil phase and mixed uniformly. PBS buffer as the water phase was mixed with the oil phase using a Scientz-IId Ultrasonic Cell Disruptor (Ningbo Scientz) to form AS03 nanoemulsions with a peptide vaccine concentration of 2 mg/mL. To enhance the solubility of peptide vaccines, the amount of squalene was increased to 2.5% to prepare F1 nanoemulsions. All other materials and procedures were the same as described in the preparation of AS03 nanoemulsions. To improve the retention of peptide vaccines at the injection site, polylactic acid (PLA) block copolymer was added to the water phase to prepare F2 emulsions based on F1 nanoemulsion prescription. The particle size of the three nanoemulsions was determined by a Malvern Zetasizer Nano ZS analyzer (Worcestershire).

### Biodistribution and retention of vaccine nanoemulsions in vivo

To evaluate the retention of peptide vaccine nanoemulsions at the injection site, Cy5-labeled peptide vaccine nanoemulsions were intramuscularly injected into the upper inner thigh of rats after hair removal. At 0, 2, 4, 6, and 8 h post injection, the rats were imaged by the IVIS Spectrum System (Caliper Corp. Waltham). Eight hours after injection, the main organs (hearts, livers, spleens, lungs, and kidneys) and the inguinal lymph nodes on one side of the injection site were collected for imaging to study the biodistribution of peptides in tissues. The fluorescence intensity of nanoemulsions at injection sites and various tissues was analyzed quantitatively with a region of interest (ROI) tool.

### RBD-binding antibody assay

RBD-binding antibodies were measured by standard enzyme-linked immunosorbent assay (ELISA) methods. 96-well ELISA plates (Nunc) were coated with wild-type RBD-His (1 μg/mL; GenScript) or mutant RBD-His (1 μg/mL; SinoBiological) in PBS overnight at 4 °C. After being blocked with 3% BSA, serial dilutions of sera were added and incubated for one hour at 37 °C with shaking. After being washed, 100 μL of Protein A-horseradish peroxidase (GenScript; 1:5000) was added to each well and incubated for one hour. After being washed, 100 μL of tetramethyl benzidine (TMB) substrate solution was added for 20 min. The reaction was terminated by 2 M H_2_SO_4_, and the absorbance value was measured at 450 nm with an Infinite F50 microplate reader (Tecan). The titer was defined as the highest dilution whose signal value remained above two times the blank signal value.

### ACE2-RBD blocking antibody detection

Antibodies that block the interaction between RBD and ACE2 were detected by the SARS-CoV-2 sVNT Kit (GenScript). Briefly, the serum samples and controls were pre-incubated with RBD-HRP to allow binding between antibodies and RBD-HRP. Then, the mixture was added to a capture plate pre-coated with the hACE2 protein. The unbound RBD-HRP and any RBD-HRP bound to non-neutralizing antibodies will be captured on the plate, while the NAbs/RBD-HRP complexes remain in the supernatant and are removed during washing. After washing steps, TMB solution is added. After adding the stop solution, this final solution was read at 450 nm in a microtiter plate reader. The absorbance of the sample was inversely dependent on the titer of the RBD-ACE2 blocking antibodies. According to the sVNT Kit, the cutoff value was set to 30%.

### Viral RNA assay

Total RNA of swabs was extracted through a High Pure Viral RNA Kit (Roche). Total RNA of lung tissue samples was extracted using TRIzol Reagent (Thermo). To determine SARS-CoV-2 RNA, a THUNDERBIRD Probe One-Step qRT-PCR Kit (TOYOBO) was used. The primers and probes used for detection included: forward primer 5’-GGGGAACTTCTCCTGCTAGAAT-3’, reverse primer 5’-CAGACATTTTGCTCTCAAGCTG-3’, and probe FAM-TTGCTGCTGCTTGACAGATT-TAMRA-3’. In each run, serial dilutions of the SARS-CoV-2 RNA reference standard (National Institute of Metrology, China) were run in parallel to calculate copy numbers in each sample.

### Live virus neutralization assay

Vero E6 cells were purchased from the American Type Culture Collection (ATCC) and cultured in Dulbecco’s Modified Eagle Medium (DMEM; Gibco) supplemented with 10% heat-inactivated fetal bovine serum (FBS; Life Technologies) and 1% penicillin/streptomycin (Invitrogen). Sera were heat-inactivated for 30 min at 56 °C. Serum dilutions were then mixed with an equal volume of viral solution containing 100 TCID_50_ for live SARS-CoV-2 (Wuhan-1). The mixture was incubated for 1 h at 37 °C. Then, 100 µL of the mixture was added in duplicate to Vero E6 cell plates. The plates were incubated for 4 days at 37 °C. After incubation, the CPE of each well was recorded under a microscope. The highest serum dilution that protected more than 50% of cells from CPE was taken as the neutralization titer.

Alternatively, quantitative reverse transcription PCR (RT-qPCR) was used to detect the neutralizing activity of antisera against live viruses. Serum dilutions were incubated with 5 μL of SARS-CoV-2 (Wuhan-1; MOI = 0.05) at 37 °C for 1 h. Then, 100 μL of antiserum-virus mixture was added to the Vero E6 cell plates. After incubation at 37 °C for 1 h, the supernatants were completely removed and replaced by fresh medium. The cell supernatants were collected and subjected to viral RNA isolation after 24 h. The viral genome copy numbers were determined by RT-qPCR with primers targeting the *S* gene.

### Pseudovirus neutralization assay

Pseudovirus neutralization assays were performed as in our previous work^36^. In brief, serum samples were serially diluted in medium after heat inactivation and then mixed with 50 μL of diluted pseudovirus. After incubation at 37 °C for 1 h, the serum-pseudovirus mixtures were transferred to wells of 96-well plates seeded 12 h earlier with 2 × 10^4^ hACE2-293T cells. Cells were assayed using the Bright-Glo™ Luciferase Assay System (Promega) after 48 h, with the relative light units (RLUs) being read on a luminometer (Promega GloMax 96). The neutralization titers were calculated as 50% inhibitory doses, expressed as the highest dilution of sample that resulted in a 50% reduction in RLUs relative to virus control wells after subtraction of background.

### T-cell immune immunophenotyping

PBMCs were isolated from cynomolgus monkeys’ blood samples by lymphocyte separation solution (Dakewei). Samples were first blocked with anti-CD16/CD32 (FcγRIII/FcγRII, 2.4G2, BD Biosciences), then incubated with surface marker antibodies for 25 min at 4 °C and then permeabilized with BD Cytofix/Cytoperm buffer before intracellular labeling antibodies were added for 30 min at 4 °C. Flow cytometry analysis was performed using ACEA NovoCyte. Data processing was done through NovoExpress software. Antibody staining of cells for flow cytometry analysis was performed following the antibody manufacturer’s recommendations. The antibodies used in T-cell immunophenotyping included FITC Mouse Anti-Human CD3ε Clone SP34 (BD Biosciences), APC Mouse Anti-Human CD28 Clone CD28.2 (BD Biosciences), PE-Cy7 Mouse Anti-Human CD45RA Clone 5H9 (BD Biosciences), and Brilliant Violet 650 anti-human CD197 (CCR7) Antibody (Biolegend).

### T-cell immune-response assay

PBMCs from cynomolgus monkeys and 40 μg/mL of LY54 or RBD were co-incubated for 8 h with brefeldin A (BFA; BD Biosciences). The staining and detection methods were the same as above. The antibodies used in intracellular cytokine staining included Hu/NHP CD3 Epsilon FITC (BD Biosciences), Hu/NHP CD4 BV421 L200 (BD Biosciences), Hu CD8 Brilliant Violet 786 (BD Biosciences), Hu IFN-Gma PercpCy5.5 (BD Biosciences), Hu TNF BV650 Mab11 (BD Biosciences), Hu IL-2 BV605 MQ1-17H12 (BD Biosciences), Hu/NHP IL-6 PE MQ2-6A3 (BD Biosciences), APC Mouse Anti-Human IL-4 (BD Biosciences), and anti-human IL-10 BV421 (BD Biosciences).

### RNA sequencing

RNA sequencing was performed by LC Sciences. Total RNA was isolated from cynomolgus monkeys’ blood samples and purified using TRIzol reagent (Invitrogen) and quantified using NanoDrop ND-1000. After cDNA library construction, sequencing was performed by Novaseq 6000 (Illumina). Based on the raw data, we analyzed immune-related signature gene levels and immune cell levels. The following groups were compared: saline vs CoVac501. Immune signature scores are defined as the mean log_2_(fold-change) among all genes in each gene signature during immune-related signature gene-level analysis. We analyzed immune cell levels using xCell^30^.

### Statistical analysis

Statistical analysis was performed using GraphPad Prism 8 Software and Origin 2019 software. Statistical methods and sample sizes in the experiments are described in each figure. *P* values < 0.05 were considered to be significant.

## Supplementary information

Supplementary Information

Supplementary Table S2

Supplementary Table S3

Supplementary Table S4

## Data Availability

All data are available in the manuscript or Supplementary Information. The raw data of RNA sequencing can be found in Supplementary Table S4. Correspondence and requests for materials should be addressed to L.G.

## References

[1] Dong E, Du H, Gardner L (2020). An interactive web-based dashboard to track COVID-19 in real time. Lancet Infect. Dis..

[2] van Dorp L (2020). Emergence of genomic diversity and recurrent mutations in SARS-CoV-2. Infect. Genet. Evol..

[3] Lauring AS, Hodcroft EB (2021). Genetic variants of SARS-CoV-2-what do they mean?. J. Am. Med. Assoc..

[4] Haynes BF (2020). Prospects for a safe COVID-19 vaccine. Sci. Transl. Med..

[5] Krammer F (2020). SARS-CoV-2 vaccines in development. Nature.

[6] Bok K, Sitar S, Graham BS, Mascola JR (2021). Accelerated COVID-19 vaccine development: milestones, lessons, and prospects. Immunity.

[7] Malonis RJ, Lai JR, Vergnolle O (2020). Peptide-based vaccines: current progress and future challenges. Chem. Rev..

[8] Nelde A, Rammensee H-G, Walz JS (2021). The peptide vaccine of the future. Mol. Cell. Proteom..

[9] Li W, Joshi MD, Singhania S, Ramsey KH, Murthy AK (2014). Peptide vaccine: progress and challenges. Vaccines.

[10] Zom GG, Khan S, Filippov DV, Ossendorp F (2012). TLR ligand-peptide conjugate vaccines: toward clinical application. Adv. Immunol..

[11] van Duin D, Medzhitov R, Shaw AC (2006). Triggering TLR signaling in vaccination. Trends Immunol..

[12] Fitzgerald KA, Kagan JC (2020). Toll-like receptors and the control of immunity. Cell.

[13] Wang X (2018). Gastric cancer vaccines synthesized using a TLR7 agonist and their synergistic antitumor effects with 5-fluorouracil. J. Transl. Med..

[14] Hu Y (2020). A novel TLR7 agonist as adjuvant to stimulate high quality HBsAg-specific immune responses in an HBV mouse model. J. Transl. Med..

[15] Wille-Reece, U. et al. HIV Gag protein conjugated to a toll-like receptor 7/8 agonist improves the magnitude and quality of Th1 and CD8+ T cell responses in nonhuman primates. *Proc. Natl. Acad. Sci. USA* **102**, 15190–15194 (2005). 10.1073/pnas.0507484102 PMC1257732 16219698

[16] Kasturi SP (2020). 3M-052, a synthetic TLR-7/8 agonist, induces durable HIV-1 envelope-specific plasma cells and humoral immunity in nonhuman primates. Sci. Immunol..

[17] Zhou P (2020). A pneumonia outbreak associated with a new coronavirus of probable bat origin. Nature.

[18] Lan J (2020). Structure of the SARS-CoV-2 spike receptor-binding domain bound to the ACE2 receptor. Nature.

[19] Piccoli L (2020). Mapping neutralizing and immunodominant sites on the SARS-CoV-2 spike receptor-binding domain by structure-guided high-resolution serology. Cell.

[20] Kreer C (2020). Longitudinal isolation of potent near-germline SARS-CoV-2-neutralizing antibodies from COVID-19 patients. Cell.

[21] Robbiani DF (2020). Convergent antibody responses to SARS-CoV-2 in convalescent individuals. Nature.

[22] Zhang BZ (2020). Mining of epitopes on spike protein of SARS-CoV-2 from COVID-19 patients. Cell Res..

[23] Lu S (2021). The immunodominant and neutralization linear epitopes for SARS-CoV-2. Cell Rep..

[24] Reynisson B, Alvarez B, Paul S, Peters B, Nielsen M (2020). NetMHCpan-4.1 and NetMHCIIpan-4.0: improved predictions of MHC antigen presentation by concurrent motif deconvolution and integration of MS MHC eluted ligand data. Nucleic Acids Res..

[25] Jespersen MC, Peters B, Nielsen M, Marcatili P (2017). BepiPred-2.0: improving sequence-based B-cell epitope prediction using conformational epitopes. Nucleic Acids Res..

[26] Aiga T (2020). Immunological evaluation of co-assembling a lipidated peptide antigen and lipophilic adjuvants: self-adjuvanting anti-breast-cancer vaccine candidates. Angew. Chem. Int. Ed. Engl..

[27] Zhu J (2015). Local administration of a novel Toll-like receptor 7 agonist in combination with doxorubicin induces durable tumouricidal effects in a murine model of T cell lymphoma. J. Hematol. Oncol..

[28] Del Giudice G, Rappuoli R, Didierlaurent AM (2018). Correlates of adjuvanticity: a review on adjuvants in licensed vaccines. Semin. Immunol..

[29] Morel S (2011). Adjuvant System AS03 containing alpha-tocopherol modulates innate immune response and leads to improved adaptive immunity. Vaccine.

[30] Aran D, Hu Z, Butte AJ (2017). xCell: digitally portraying the tissue cellular heterogeneity landscape. Genome Biol..

[31] Supasa P (2021). Reduced neutralization of SARS-CoV-2 B.1.1.7 variant by convalescent and vaccine sera. Cell.

[32] Mahase E (2021). Covid-19: Novavax vaccine efficacy is 86% against UK variant and 60% against South African variant. BMJ.

[33] Madhi SA (2021). Efficacy of the ChAdOx1 nCoV-19 Covid-19 vaccine against the B.1.351 variant. N. Engl. J. Med..

[34] Zhou D (2021). Evidence of escape of SARS-CoV-2 variant B.1.351 from natural and vaccine-induced sera. Cell.

[35] Thomson EC (2021). Circulating SARS-CoV-2 spike N439K variants maintain fitness while evading antibody-mediated immunity. Cell.

[36] He X (2021). A human cell-based SARS-CoV-2 vaccine elicits potent neutralizing antibody responses and protects mice from SARS-CoV-2 challenge. Emerg. Microbes Infect..

[37] Gobeil SM (2021). Effect of natural mutations of SARS-CoV-2 on spike structure, conformation, and antigenicity. Science.

[38] Wang WB (2021). E484K mutation in SARS-CoV-2 RBD enhances binding affinity with hACE2 but reduces interactions with neutralizing antibodies and nanobodies: binding free energy calculation studies. J. Mol. Graph. Model..

[39] Wheatley AK (2021). Immune imprinting and SARS-CoV-2 vaccine design. Trends Immunol..

[40] Wibmer CK (2021). SARS-CoV-2 501Y.V2 escapes neutralization by South African COVID-19 donor plasma. Nat. Med..

[41] Shi R (2020). A human neutralizing antibody targets the receptor-binding site of SARS-CoV-2. Nature.

[42] Zost SJ (2020). Potently neutralizing and protective human antibodies against SARS-CoV-2. Nature.

[43] Yuan M (2020). Structural basis of a shared antibody response to SARS-CoV-2. Science.

[44] Yuan M (2020). A highly conserved cryptic epitope in the receptor binding domains of SARS-CoV-2 and SARS-CoV. Science.

[45] Wrapp D (2020). Structural basis for potent neutralization of betacoronaviruses by single-domain camelid antibodies. Cell.

[46] Huo J (2020). Neutralization of SARS-CoV-2 by destruction of the prefusion spike. Cell Host Microbe.

[47] Li Y (2020). Linear epitopes of SARS-CoV-2 spike protein elicit neutralizing antibodies in COVID-19 patients. Cell. Mol. Immunol..

[48] McCallum M (2021). N-terminal domain antigenic mapping reveals a site of vulnerability for SARS-CoV-2. Cell.

[49] Chi X (2020). A neutralizing human antibody binds to the N-terminal domain of the Spike protein of SARS-CoV-2. Science.

[50] Delgado MF (2009). Lack of antibody affinity maturation due to poor Toll-like receptor stimulation leads to enhanced respiratory syncytial virus disease. Nat. Med..

[51] Chan JF-W (2020). Genomic characterization of the 2019 novel human-pathogenic coronavirus isolated from a patient with atypical pneumonia after visiting Wuhan. Emerg. Microbes Infec..

[52] Diebold SS, Kaisho T, Hemmi H, Akira S, Reis e Sousa C (2004). Innate antiviral responses by means of TLR7-mediated recognition of single-stranded RNA. Science.

[53] Bunders MJ, Altfeld M (2020). Implications of sex differences in immunity for SARS-CoV-2 pathogenesis and design of therapeutic interventions. Immunity.

[54] Mueller AL, McNamara MS, Sinclair DA (2020). Why does COVID-19 disproportionately affect older people?. Aging.

[55] Poulas K, Farsalinos K, Zanidis C (2020). Activation of TLR7 and innate immunity as an efficient method against COVID-19 pandemic: imiquimod as a potential therapy. Front. Immunol..

[56] Iwasaki A, Omer SB (2020). Why and how vaccines work. Cell.

