# Supplementary material for: CoVac501, a self-adjuvanting peptide vaccine conjugated with TLR7 agonists, against SARS-CoV-2 induces protective immunity
Source: Cell Discov. 2022 Feb 1;8:9. doi: 10.1038/s41421-021-00370-2 (PMC8803929; doi:10.1038/s41421-021-00370-2)
Supplement: Supplementary file 1 — Supplementary Information [file 41421_2021_370_MOESM1_ESM.pdf]

Supplementary Information

Title: CoVac501, a self-adjuvanting peptide vaccine conjugated with TLR7 agonists, against SARS-CoV-2 induces protective immunity

Authors: Yiru Long[1,2], Jianhua Sun[1,2], Tian-Zhang Song[2,3], Tingting Liu[1], Feng Tang[1,2], Xinxin Zhang[1,2], Longfei Ding[4], Yunqiu Miao[1,2], Weiliang Zhu[1,2], Xiaoyan Pan[2,5], Qi An[6], Mian Qin[7], Xiankun Tong[1,2], Xionghua Peng[1], Pan Yu[1], Peng Zhu[1], Jianqing Xu[4], Xiaoyan Zhang[4], Yachun Zhang[6], Datao Liu[8], Ben Chen[8], Huilin Chen[8], Leike Zhang[2,5], Gengfu Xiao[2,5], Jianping Zuo[1,2], Wei Tang[1,2], Ji Zhou[9,10], Heng Li[1,2], Zhijian Xu[1,2], Hong-Yi Zheng[2,3], Xin-Yan Long[2,3], Qiuping Qin[1*], Yong Gan[1,2*], Jin Ren[1,2*], Wei Huang[1,2,11*], Yong-Tang Zheng[2,3*], Guangyi Jin[9,10*], Likun Gong[1,2,7*]

This file includes:
Fig. S1 to Fig. S20
Table S1

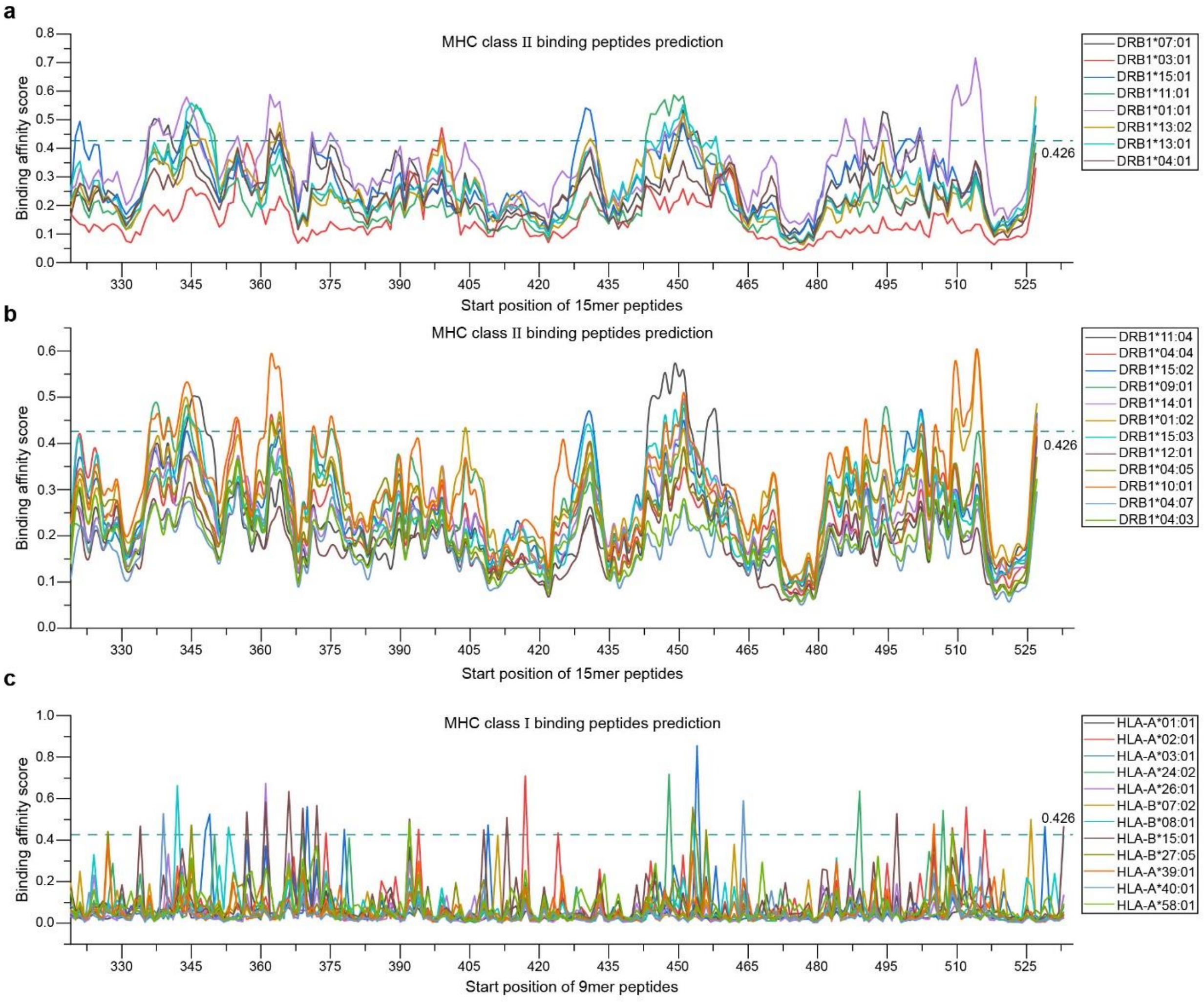

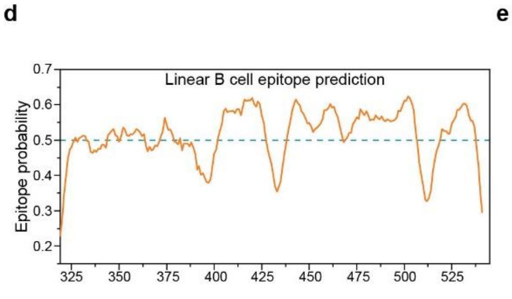

e

Immunological characteristics of candidate peptides

Peptide ID	Position	Potential binding HLA II alleles
P67	351-378	HLA-DRB1: 07:01, 15:01, 11:01, 01:01, 13:02, 13:01, 04:01, 11:04, 04:04, 15:02, 09:01, 14:01, 01:02, 15:03, 04:05, 10:01, 04:07, 04:03<br>HLA-DRB3: 01:01, 02:02<br>HLA-DRB4: 01:01<br>HLA-DRB5: 01:01
LY54	455-508	HLA-DRB1:<br>07:01, 01:01, 13:02, 13:01, 04:01, 11:04, 15:02, 09:01, 14:01, 01:02, 04:05, 10:01<br>HLA-DRB3: 01:01,02:02<br>HLA-DRB4: 01:01<br>HLA-DRB5: 01:01

Fig. S1. Immunogenicity prediction and candidate peptide selection for RBD. a, b The HLA class II binding peptides of RBD for major HLA class II types were predicted by NetMHCIIpan-4.0. Graphs were created with Origin 2019 software. **c** The HLA class I binding peptides of RBD for major HLA class I types were predicted by NetMHCpan-4.1. Graph was created with Origin 2019 software. **d** B-cell linear epitopes of RBD were predicted by BepiPred-2.0. Graph was created with Origin 2019 software. **e** The table shows the potential binding HLA II alleles for LY54 and P67.

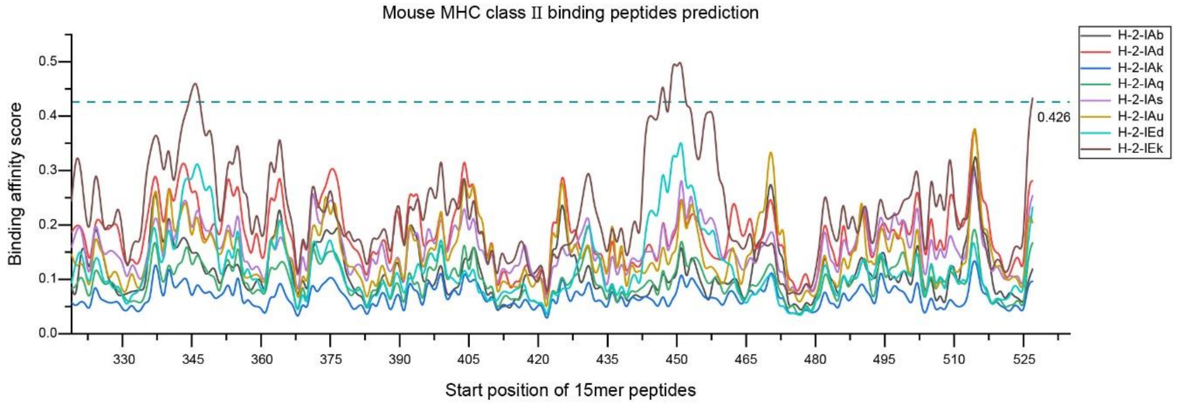

Fig. S2. Prediction of MHC class II binding peptides of RBD for major mouse MHC class II types. The MHC class II binding peptides of RBD for major mouse MHC class II types were predicted by NetMHCIIpan-4.0. Graphs were created with Origin 2019 software.

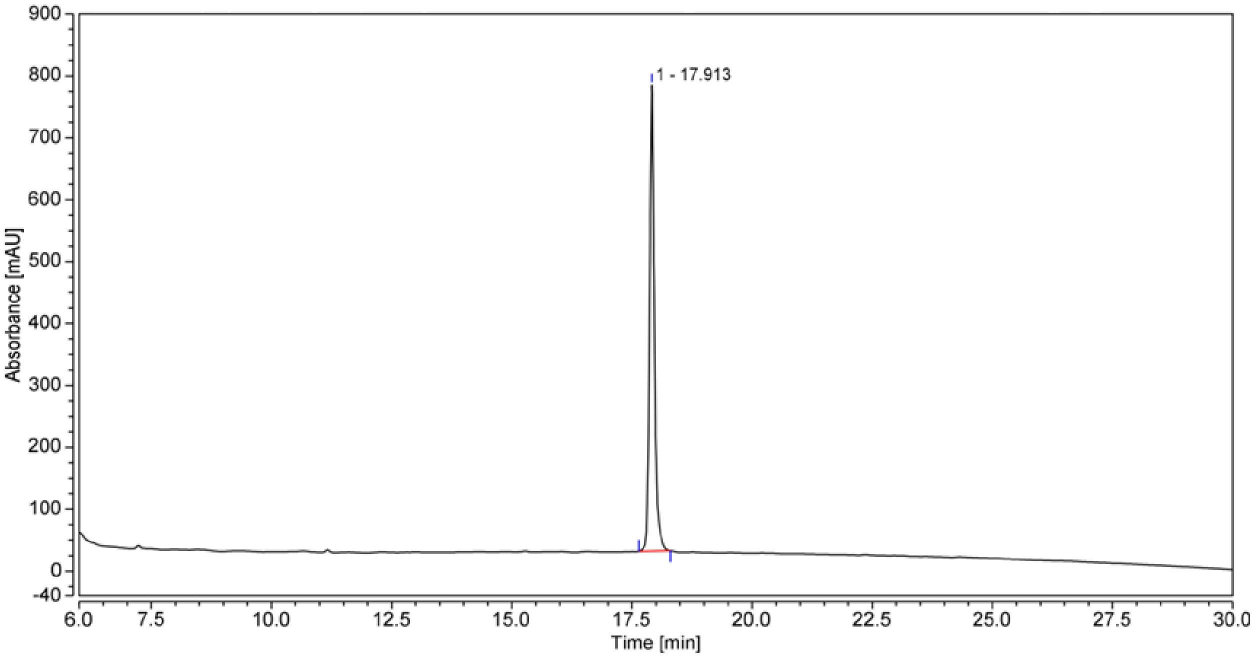

Fig. S3. HPLC profile of purified LY54-101. The LY54-101 was purified by preparative C18 column and was obtained as a white powder after lyophilization. The purity of LY54-101 was identified by HPLC.

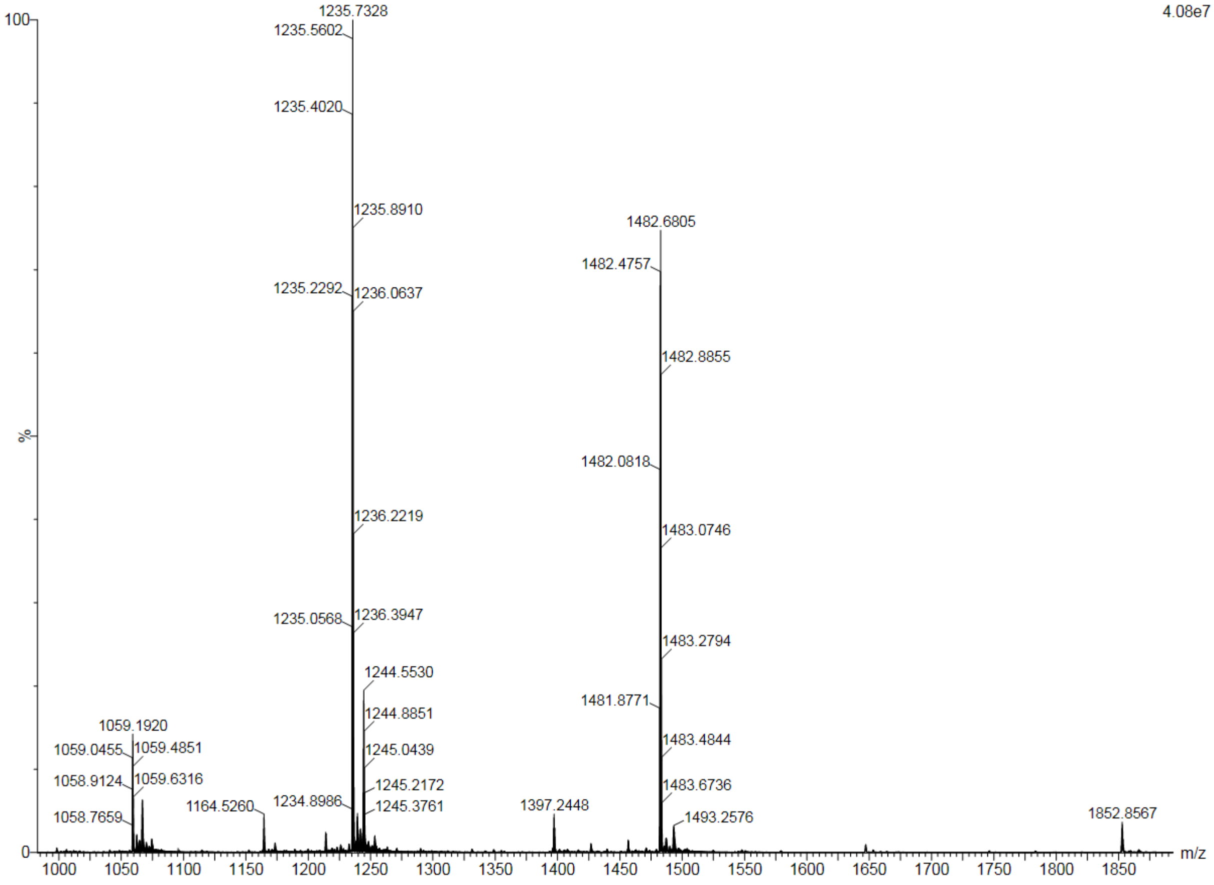

Fig. S4. HRMS profile of purified LY54-101. The purified LY54-101 was identified by HRMS.

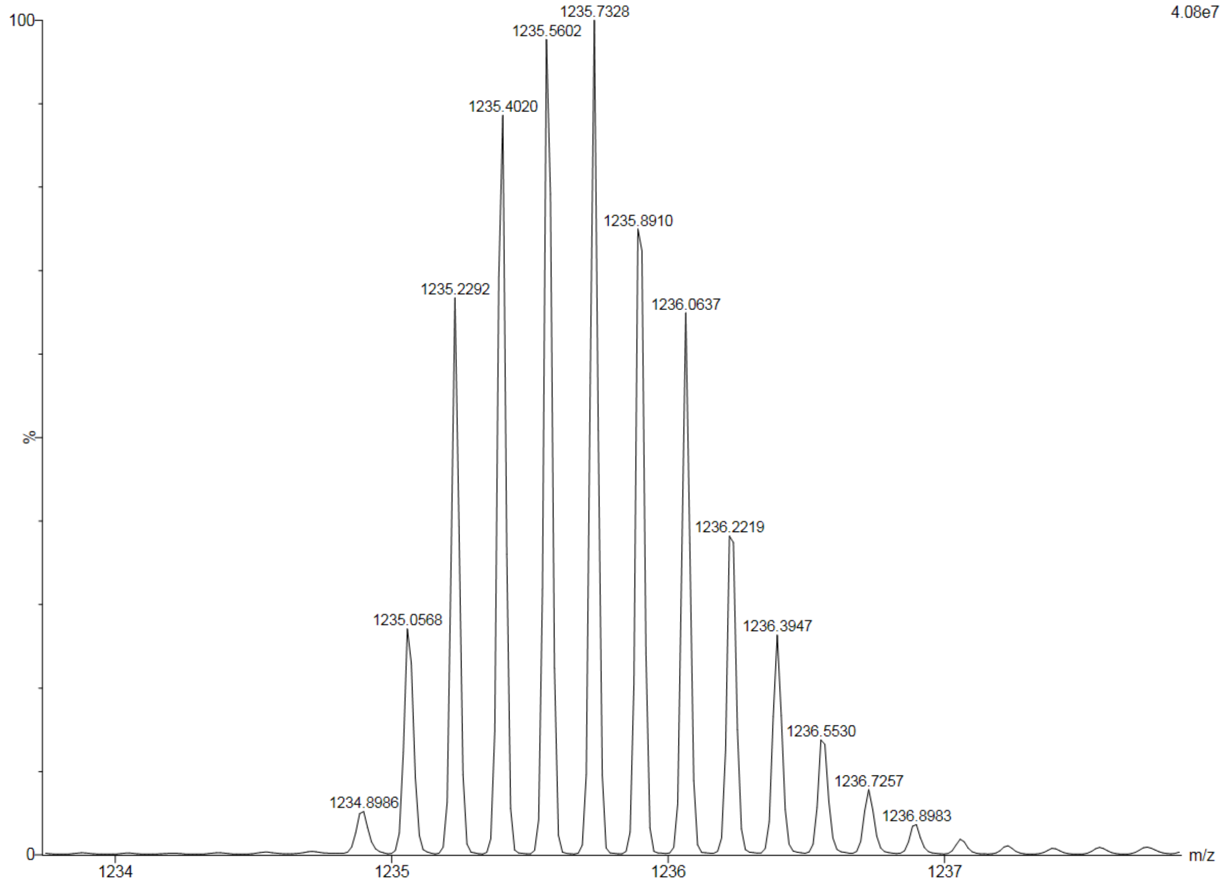

Fig. S5. HRMS profile of purified LY54-101 (magnified [M + 6H]$^{6+}$ peaks).

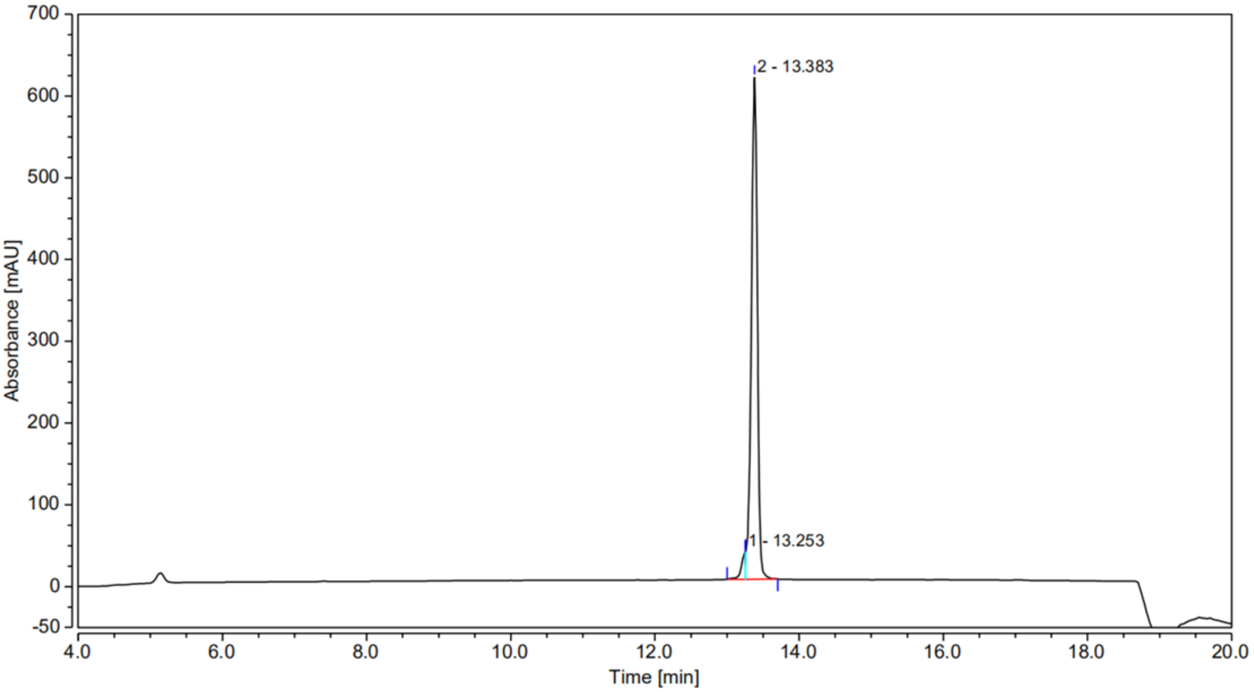

Fig. S6. HPLC profile of purified P67-101. The P67-101 was purified by preparative C18 column chromatography to become a white powder after lyophilization. The purity of P67-101 was identified by HPLC.

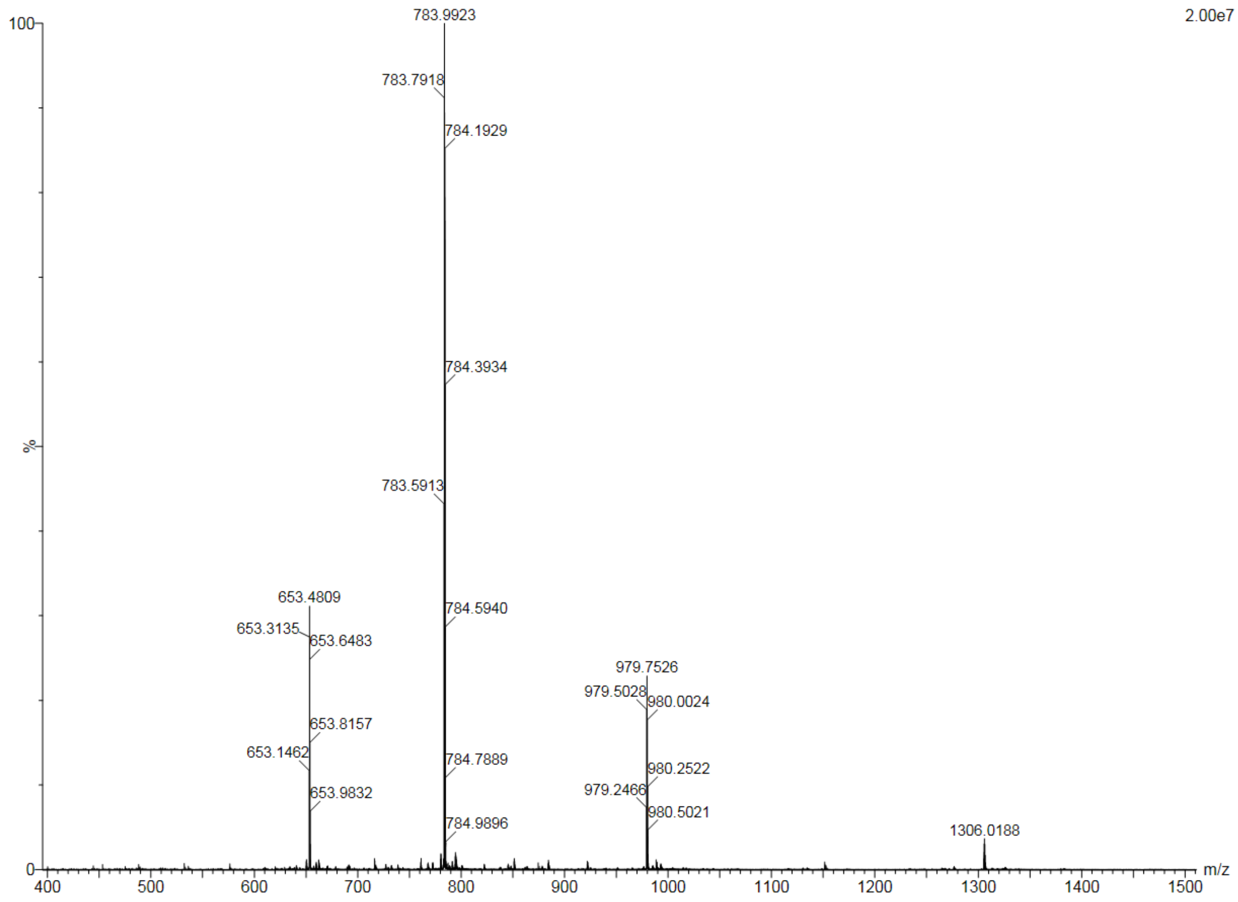

Fig. S7. HRMS profile of purified P67-101. The purified P67-101 was identified by HRMS.

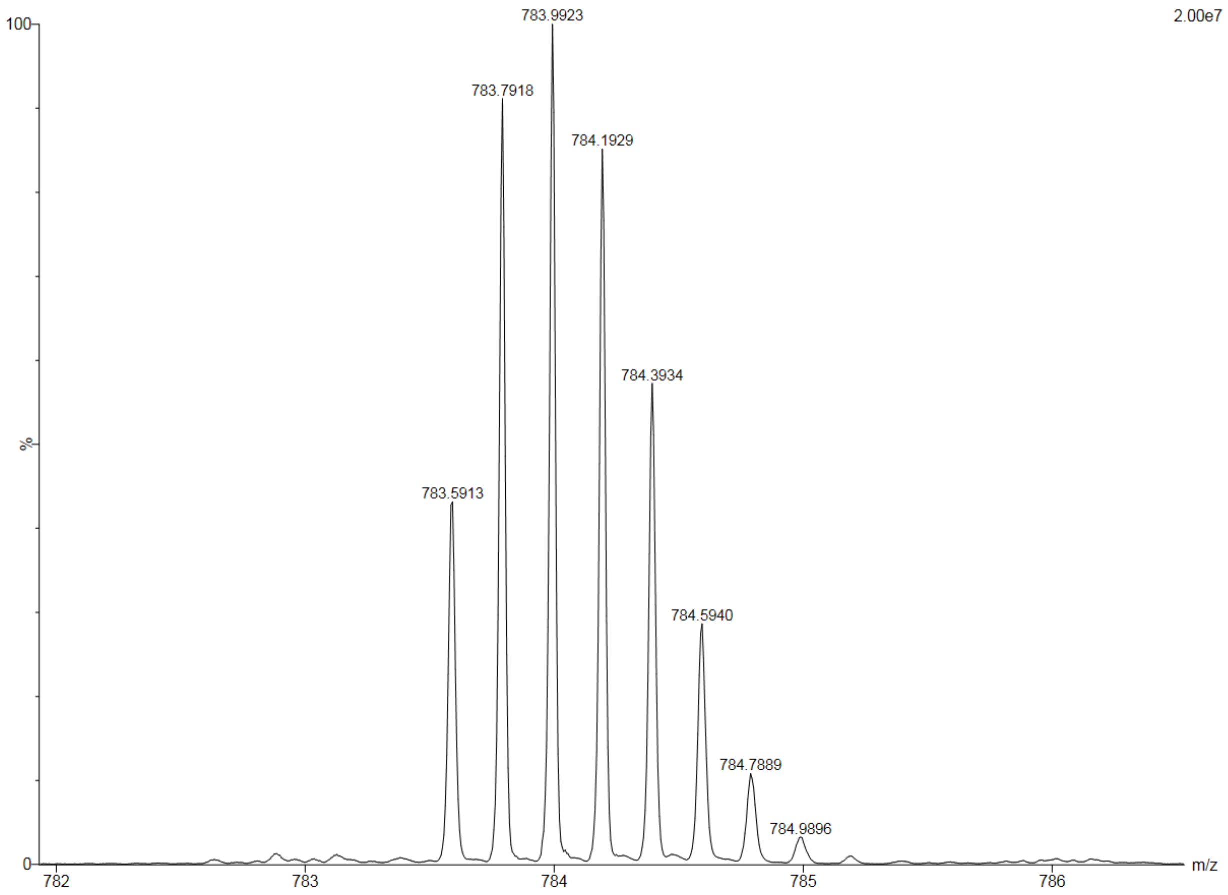

Fig. S8. HRMS profile of purified P67-101 (magnified [M + 5H]$^{5+}$ peaks).

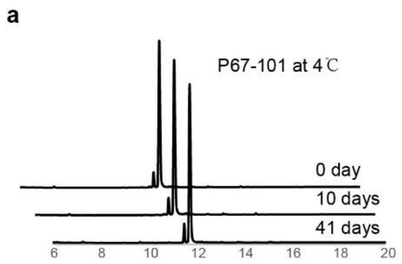

Fig. S9. Stability analysis of P67-101 (in CoVac501). a-c HPLC profiles of stability analysis of P67-101 for 0 days, 10 days and 41 days at 4℃ (**a**), 25℃ (**b**) and 40℃ (**c**). The horizontal axis represents the retention of time (min) and the vertical axis represents the absorbance (mAU).

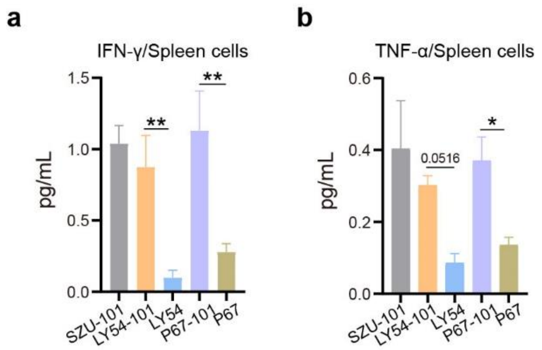

Fig. S10. Immunostimulatory effect of LY54-101 and P67-101. a, b Spleen cells obtained from cynomolgus monkeys were treated with SZU-101, LY54-101, LY54, P67-101, and P67 respectively. IFN-γ (**a**) and TNF-α (**b**) were detected by electrochemiluminescence (ECL) immunoassays. ** $P < 0.01$ and * $P < 0.05$ as determined by one-way ANOVA with multiple comparison tests. All data are presented as the mean ± SEM.

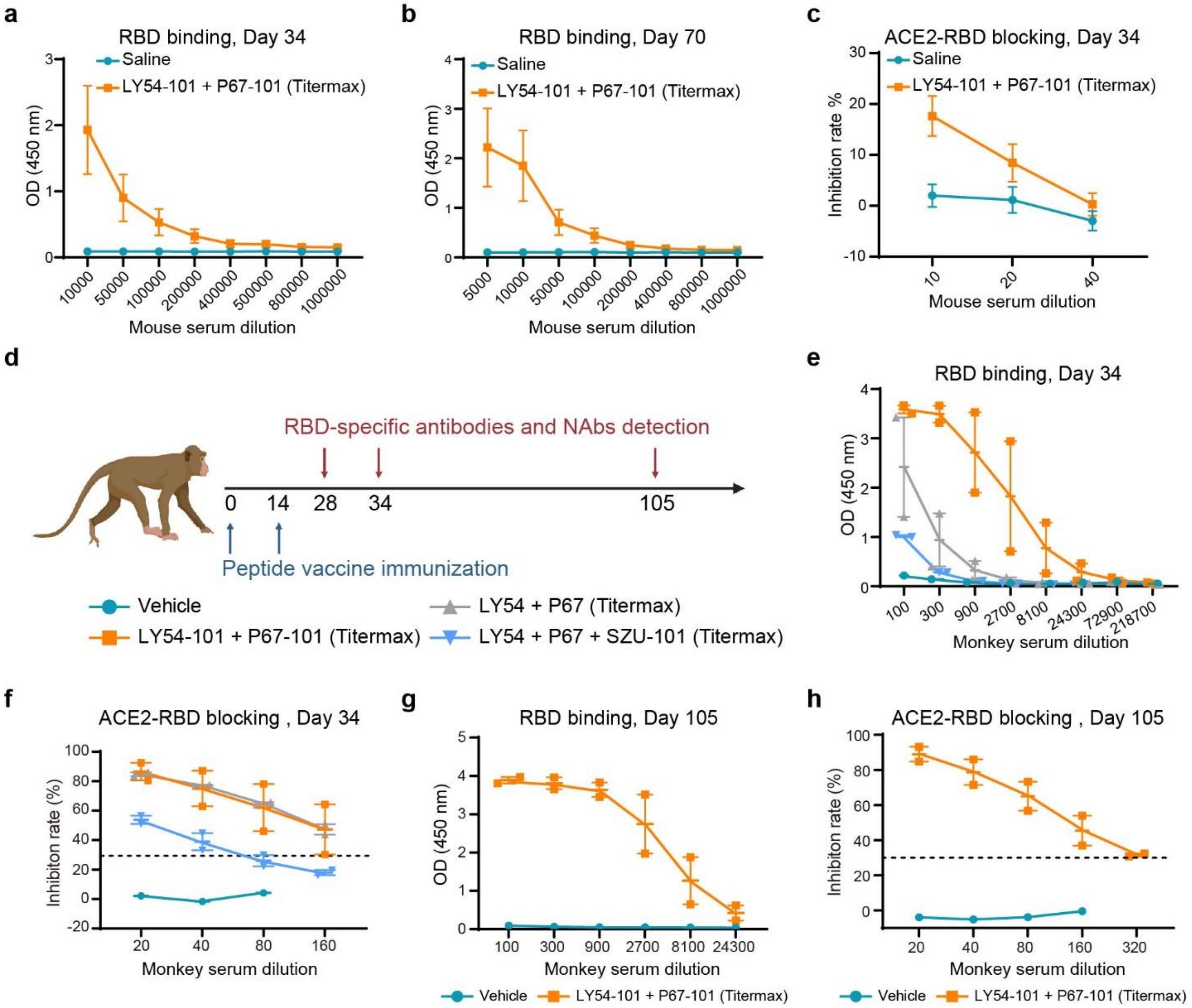

Fig. S11. Immunogenicity of LY54-101 and P67-101. a-c The C57BL/6 mice (n = 5) were immunized with LY54-101 and P67-101 in the presence of Titermax for three once-daily intramuscular injections on days 0, 7 and 14, respectively. Serum samples were collected from the mice 34 days (**a, c**) and 70 days (**b**) after the first dose of vaccine and the levels of IgG-type binding antibodies against the recombinant RBD protein and ACE2-RBD blocking activity were determined for different serum dilutions using ELISAs. **d** Schematic diagram of immunization, sample collection and antibody related detection in cynomolgus monkeys. **e, f** The cynomolgus monkeys (n = 2) were immunized in the following groups: Vehicle, LY54-101 + P67-101, LY54 + P67 and LY54 + P67 + SZU-101 in the presence of Titermax for two once-daily intramuscular injections on days 0 and 14, respectively. Sera were collected from the monkeys 34 days after the first dose of vaccine and the levels of RBD-specific antibodies (**e**) and ACE2-RBD blocking activity (**f**) were determined for different serum dilutions using ELISAs. A 30% inhibition rate is the cut-point for a positive titer of ACE2-RBD blocking antibodies. **g, h** Sera were collected from the monkeys 105 days after the first dose of vaccine and the levels of RBD-specific binding antibodies (**g**) and ACE2-RBD blocking activity (**h**) were determined. All data are presented as the mean ± SEM.

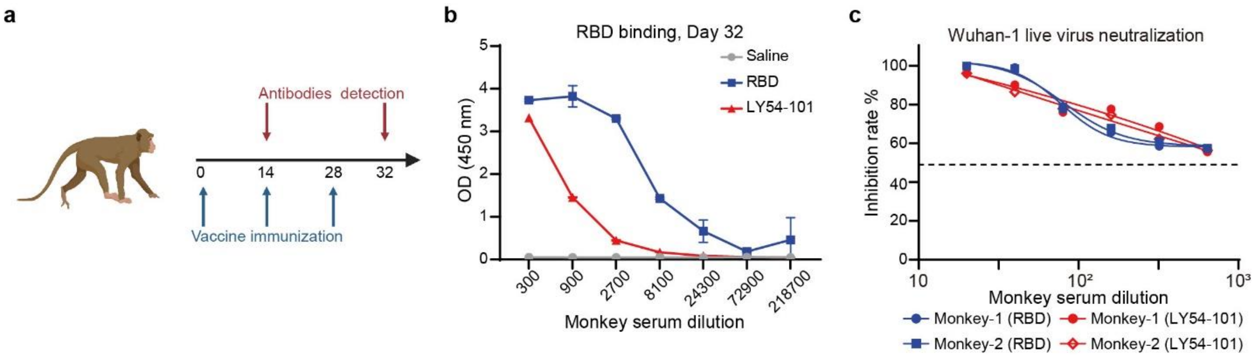

Fig. S12. Comparison of the immunogenicity of LY54-101 and RBD. a Schematic diagram of immunization, sample collection and antibody related detection in cynomolgus monkeys. **b**, **c** The cynomolgus monkeys of different groups (n = 2/group) were immunized with Saline, RBD and LY54-101, respectively, in the presence of Titermax for three once-daily intramuscular injections on days 0, 14 and 28. Sera were collected from the monkeys 32 days after the first dose of vaccination and the levels of RBD-specific binding antibodies (**b**) were determined for different serum dilutions using ELISAs. The neutralization activity for Wuhan-1 live virus (**c**) was analyzed through RT-qPCR.

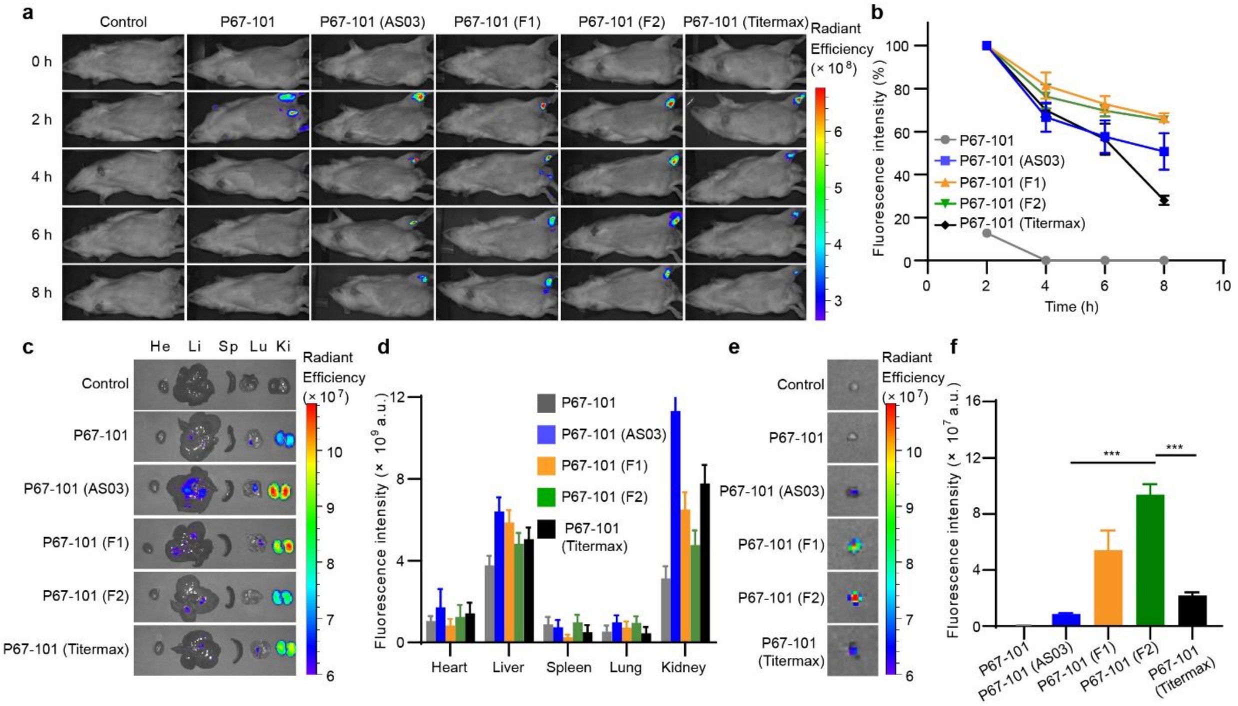

Fig. S13. Biodistribution and retention of P67-101 nanoemulsions in vivo. a Real-time in vivo fluorescence images of rats after intramuscular injection of Cy5 labeled P67-101 nanoemulsions. **b** Monitoring of the kinetics of P67-101 nanoemulsion retention at injection sites within 8 h. (n = 3; mean ± SD). **c** Fluorescence images of organs excised at 8 h postinjection. He, hearts; Li, livers; Sp, spleens; Lu, lungs; Ki, kidneys. **d** Biodistribution of Cy5 labeled P67-101 nanoemulsions in main organs excised from rats at 8 h postinjection. (n = 3; mean ± SD). **e** Fluorescence images of lymph nodes excised at 8 h postinjection. **f** Fluorescence intensity of Cy5 labeled P67-101 nanoemulsions in lymph nodes excised from rats at 8 h postinjection. (n = 3; mean ± SD), *** $P < 0.001$ as determined by one-way ANOVA with multiple comparison tests.

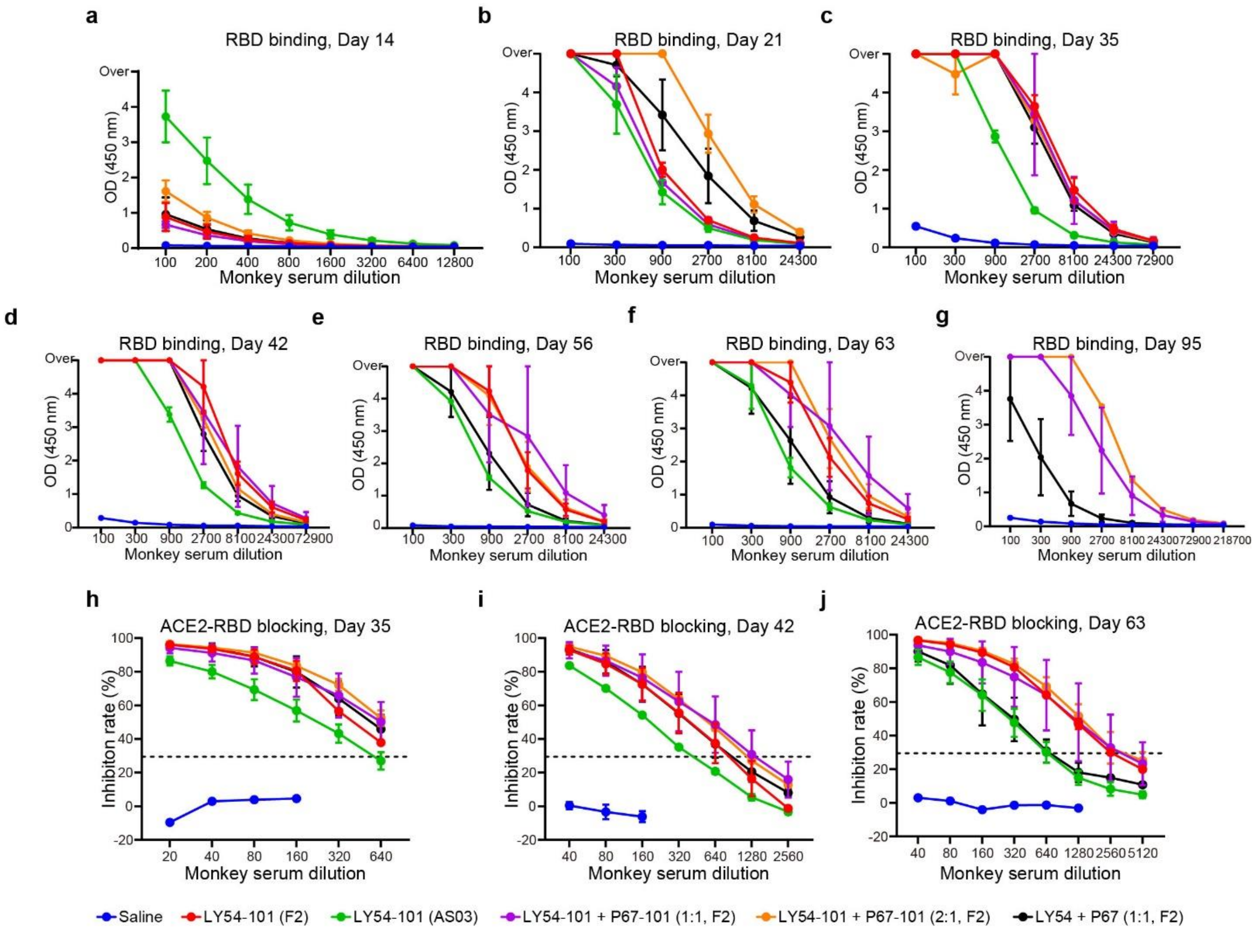

Fig. S14. Levels of RBD binding antibodies and ACE2-RBD blocking activity in vaccinated cynomolgus monkeys. a-g The cynomolgus monkeys (n = 2) of different groups were immunized with peptide vaccines containing saline, LY54-101 (F2), LY54-101 (AS03), LY54-101 + P67-101 (1:1, F2), LY54-101 + P67-101 (2:1, F2) and LY54 + P67 (1:1, F2), respectively, for three doses on days 0, 14 and 28. Sera were collected from the monkeys 14 (**a**), 21 (**b**), 35 (**c**), 42 (**d**), 56 (**e**), 63 (**f**) and 95 (**g**) days after the first dose of vaccine and the levels of RBD-specific binding antibodies were tested for different serum dilutions using the ELISA. **h-j** Sera were collected from the monkeys 35 (**h**), 42 (**i**) and 63 (**j**) days after the first dose of the vaccination and ACE2-RBD blocking antibodies were determined for different serum dilutions using ELISAs. All data are presented as the mean ± SEM.

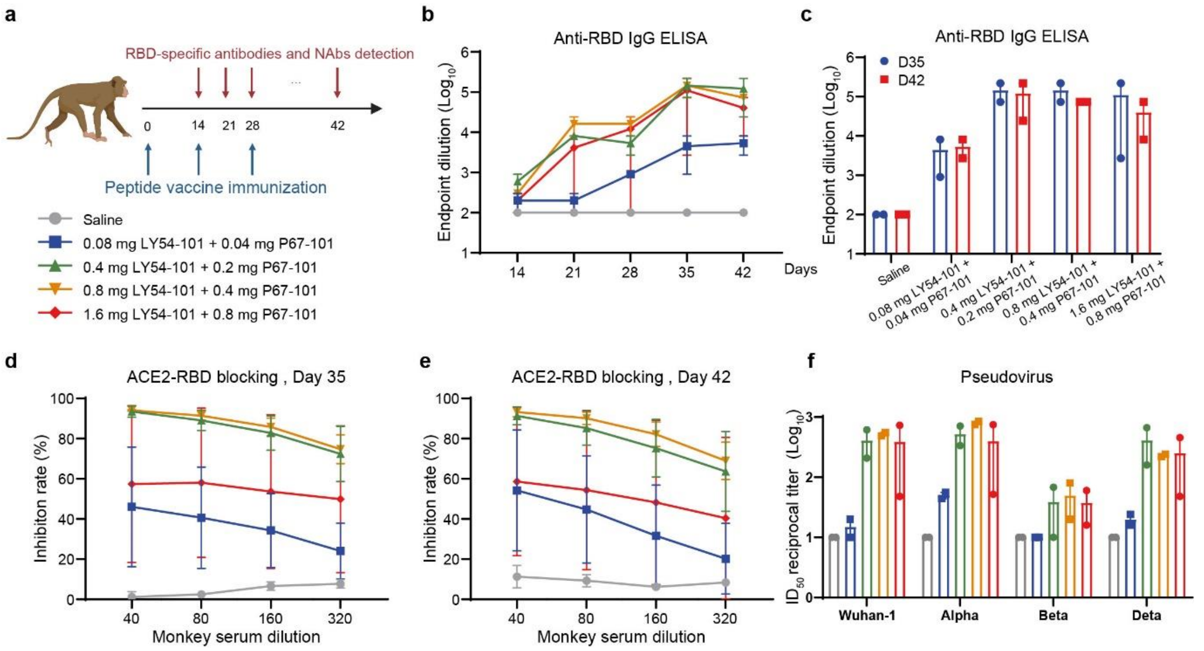

Fig. S15. Multi-dose evaluation study for CoVac501. a Schematic diagram of immunization, sample collection and antibody detection in cynomolgus monkeys. The cynomolgus monkeys of different groups (n = 2/group) were immunized with peptide vaccines containing saline, 0.08 mg LY54-101 + 0.04 mg P67-101, 0.4 mg LY54-101 + 0.2 mg P67-101, 0.8 mg LY54-101 + 0.4 mg P67-101, and 1.6 mg LY54-101 + 0.8 mg P67-101, respectively, for three doses on days 0, 14 and 28. **b, c** Sera were collected from the monkeys and the levels of RBD-binding antibodies were determined for different serum dilutions using ELISA methods. **d, e** Sera were collected from the monkeys 35 (**d**) and 42 (**e**) days after the first dose of vaccination and ACE2-RBD blocking antibodies were tested for different serum dilutions using ELISAs. **f** The neutralization of sera from cynomolgus monkeys 35 days after the first dose of vaccination for SARS-CoV-2 pseudovirus. All data are presented as the mean ± SEM.

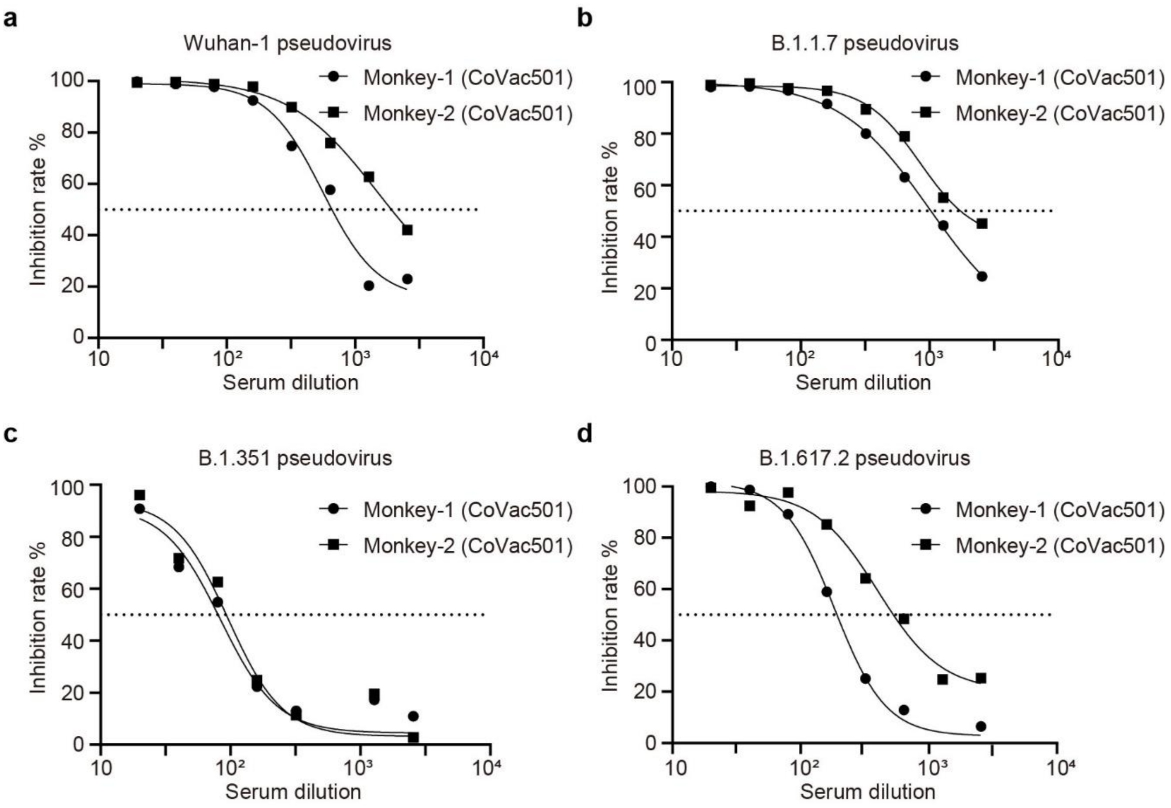

Fig. S16. The neutralization of sera from CoVac501 vaccinated monkeys for SARS-CoV-2 pseudovirus. The neutralization of sera from CoVac501 vaccinated cynomolgus monkeys 63 days after the first dose of vaccination for SARS-CoV-2 pseudovirus (Wuhan-1, B.1.1.7, B.1.351, and B.1.617.2).

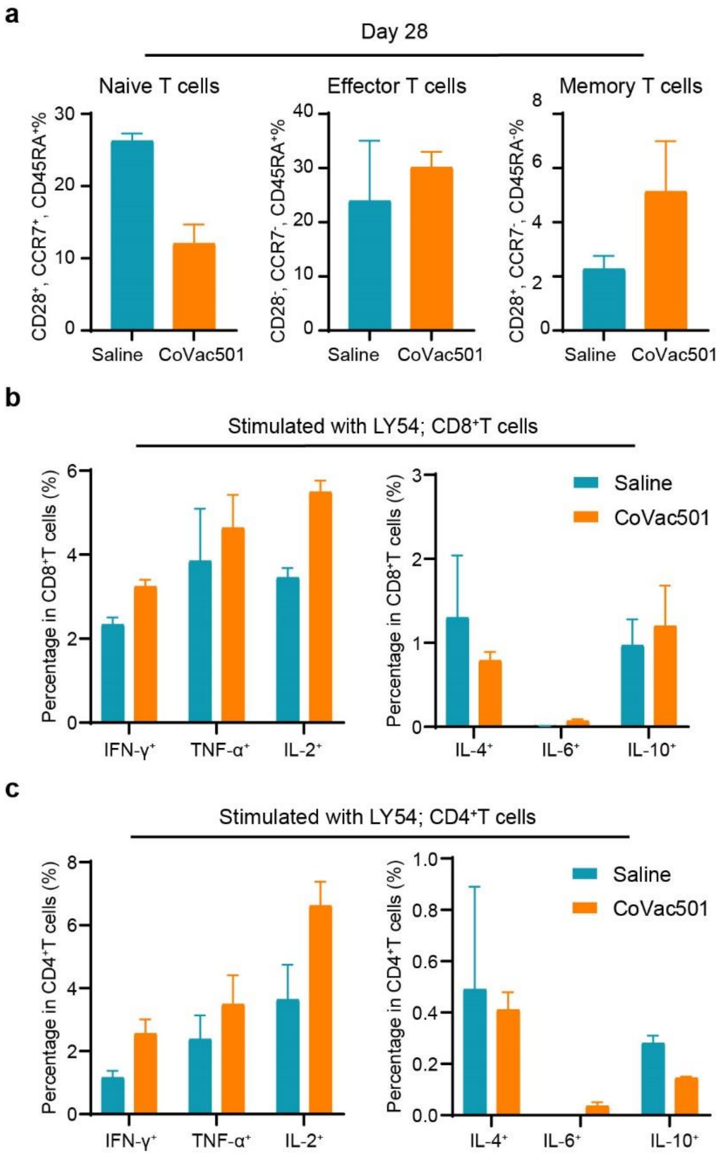

Fig. S17. CoVac501 induced Th1-baised responses and T-cell immune memory in cynomolgus macaques. a PBMCs were collected from the monkeys 28 days after the first dose of vaccination and naive T cells ($CD28^+$, $CCR7^+$ and $CD45RA^+$), effector T cells ($CD28^-$, $CCR7^-$ and $CD45RA^+$) and memory T cells ($CD28^+$, $CCR7^-$ and $CD45RA^-$) were analyzed by flow cytometry. **b, c** PBMCs were collected from the monkeys 70 days after the first dose of vaccination and IFN-γ^+, TNF-α^+, IL-2^+, IL-4^+, IL-6^+ and IL-10^+ intracellular cytokine staining assays by flow cytometry for $CD8^+$ T (**b**) and $CD4^+$ T (**c**) cells in response to LY54 after eight hours antigen stimulation. All data are presented as the mean ± SEM.

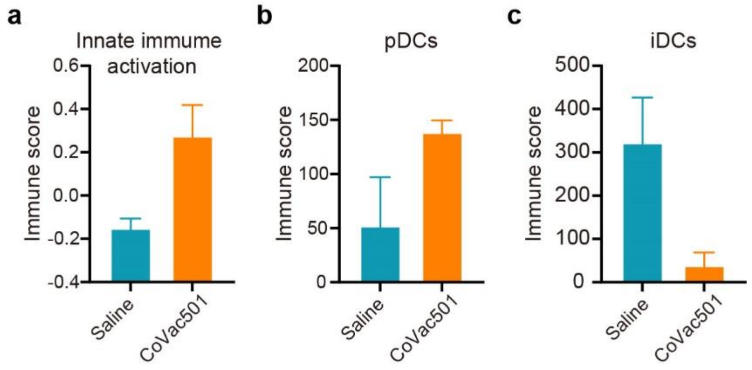

Fig. S18. RNA sequencing results of innate immune activation. a-c RNA sequencing results were analyzed for levels of immune cells and immune processes through xCell or Molecular Signatures Database. All data are presented as the mean ± SEM.

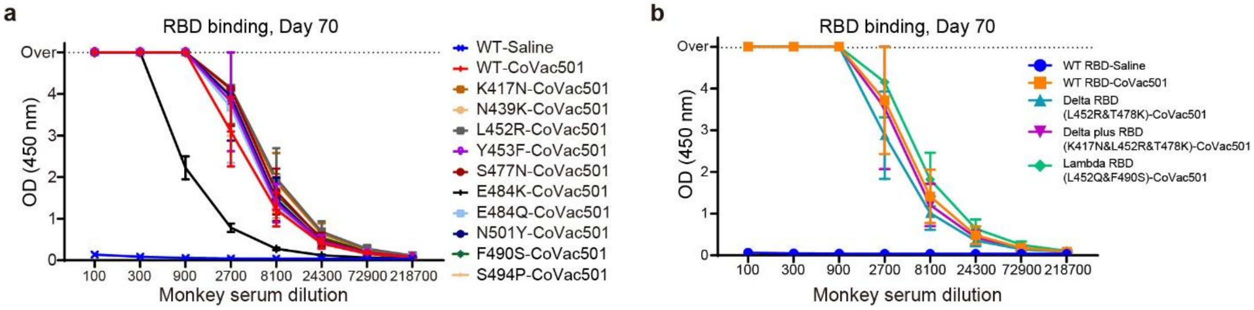

Fig. S19. Antibodies levels of sera from CoVac501 vaccinated monkeys (day 70) against RBD mutations. a, b The cynomolgus monkeys (n = 2) were immunized with saline or CoVac501 for three doses at days 0, 14 and 28. Sera were collected from the monkeys 70 days after the first dose of vaccine and the levels of RBD single mutations (**a**) and combined mutations (**b**) binding antibodies were tested for different serum dilutions using ELISAs. All data are presented as the mean ± SEM.

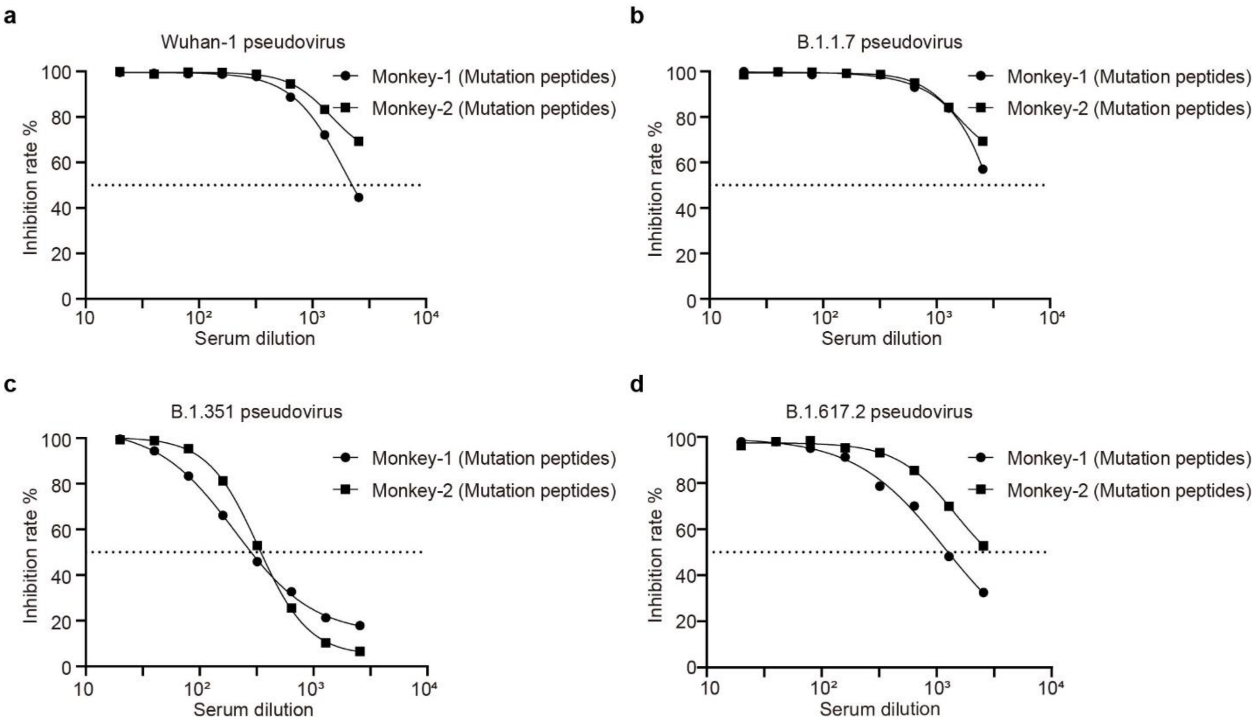

Fig. S20. The neutralization of sera from monkeys vaccinated with mutation peptides for SARS-CoV-2 pseudovirus. The neutralization of sera from cynomolgus monkeys vaccinated with mutation peptides 21 days after the first dose of vaccine for SARS-CoV-2 pseudovirus (Wuhan-1, B.1.1.7, B.1.351, and B.1.617.2).

Table S1
Stability analysis for F2 nanoemulsion.

Storage conditions	Storage time (days)				
	0	7	15	30	60
4℃ (nm)	116.3	116.9	117.1	116.5	116.8
40℃ (nm)	116.3	117.1	116.7	117.2	116.8
